# Bacterial community composition and *fhs* profiles of low- and high-ammonia biogas digesters reveal novel syntrophic acetate-oxidising bacteria

**DOI:** 10.1186/s13068-016-0454-9

**Published:** 2016-02-27

**Authors:** Bettina Müller, Li Sun, Maria Westerholm, Anna Schnürer

**Affiliations:** Department of Microbiology, Uppsala BioCenter, Swedish University of Agricultural Sciences, Box 7025, 750 07 Uppsala, Sweden

**Keywords:** Acetogens, Formyltetrahydrofolate synthetase, Syntrophic acetate oxidation, Ammonia inhibition, Illumina amplicon sequencing, Biogas production, Anaerobic degradation

## Abstract

**Background:**

Syntrophic acetate oxidation (SAO) is the predominant pathway for methane production in high ammonia anaerobic digestion processes. The bacteria (SAOB) occupying this niche and the metabolic pathway are poorly understood. Phylogenetic diversity and strict cultivation requirements hinder comprehensive research and discovery of novel SAOB. Most SAOB characterised to date are affiliated to the physiological group of acetogens. Formyltetrahydrofolate synthetase is a key enzyme of both acetogenic and SAO metabolism. The encoding *fhs* gene has therefore been identified as a suitable functional marker, using a newly designed primer pair. In this comparative study, we used a combination of terminal restriction fragment length polymorphism profiling, clone-based comparison, qPCR and Illumina amplicon sequencing to assess the bacterial community and acetogenic sub-community prevailing in high- and low-ammonia laboratory-scale digesters in order to delineate potential SAOB communities. Potential candidates identified were further tracked in a number of low-ammonia and high-ammonia laboratory-scale and large-scale digesters in order to reveal a potential function in SAO.

**Results:**

All methodical approaches revealed significant changes in the bacterial community composition concurrently with increasing ammonia and predominance of SAO. The acetogenic community under high ammonia conditions was revealed to be generally heterogeneous, but formed distinct phylogenetic clusters. The clusters differed clearly from those found under low-ammonia conditions and represented an acetogenic assemblage unique for biogas processes and recurring in a number of high-ammonia processes, indicating potential involvement in SAO.

**Conclusions:**

The phylogenetic affiliation and population dynamics observed point to a key community, belonging mainly to the Clostridia class, in particular to the orders Clostridiales and Thermoanaerobacterales, which appear to specialise in SAO rather than being metabolically versatile. Overall, the results reported here provide evidence of functional importance of the bacterial families identified in high-ammonia systems and extend existing knowledge of bacterial and acetogenic assemblages at low and high ammonia levels. This information will be of help in monitoring and assessing the impacts on the SAOB community in order to identify characteristics of robust and productive high ammonia biogas processes.

**Electronic supplementary material:**

The online version of this article (doi:10.1186/s13068-016-0454-9) contains supplementary material, which is available to authorized users.

## Background

In methanogenic environments, including constructed biogas digesters, acetate is a significant precursor for methane [1–2]. Consequently, acetate-degrading microorganisms play an important role in such environments. Methanogenesis from acetate can be performed by two different mechanisms, direct cleavage by aceticlastic methanogenesis, conducted by species belonging to Methanosarcina or Methanosaeta, or syntrophic acetate oxidation (SAO) [3]. The latter is performed by syntrophic acetate-oxidising bacteria (SAOB), which oxidise acetate to CO_2_ and H_2_/formate. This reaction is dependent on instant H_2_ removal by hydrogenotrophic methanogens, with subsequent production of CH_4_. Thermodynamic constraints predict an advantage for aceticlastic methanogenesis over acetate oxidation [4]. However, certain conditions, such as high ammonia concentrations, selectively inhibit aceticlastic methanogenesis [5, 6] and thus improve the competitiveness of SAO [7–13]. Acetate concentration, dilution rate and methanogenic community composition are other factors suggested to promote the contribution of SAO to methane production [14–18]. In deep subsurface oil reservoirs, the CO_2_ partial pressure has also been shown to influence the methanogenic pathway [19].

Very little is known about the diversity, size and dynamics of the SAOB communities in all the environments mentioned, and knowledge regarding their metabolic flexibility is lacking. Only a restricted number of SAOB have been isolated and characterised to date. They are *Pseudothermotoga lettingae*, *Thermacetogenium phaeum*, *Clostridium ultunense*, *Syntrophaceticus schinkii* and *Tepidanaerobacter acetatoxydans* [20–24]. Based on the 16S rRNA gene, these syntrophs are mainly distantly related and display substantial differences in substrate utilisation and cultivation traits in pure culture conditions. The observed acetogenic metabolism affiliates the majority of the SAOB to the group of acetogens, which are characterised by the Wood–Ljungdahl (WL) pathway as a common metabolic feature [25].

To date, two pathways have been suggested for syntrophic acetate oxidation. The first employs a reversed WL pathway, as indicated by genome-guided analysis and enzyme activity studies in case of the thermophilic *T. phaeum*, and mesophilic *C. ultunense* [26–28]. The mesophilic *T. acetatoxydans* harbours a truncated WL pathway [29]. The second pathway has been suggested for a hypermesophilic *Mesotoga* community and the thermophilic SAOB *P. lettingae* [30]. This pathway circumvents the carbonyl branch of the WL pathways by combining the glycine cleavage system with the methyl branch of the WL pathway. In the case of the mesophilic *S. schinkii*, a previously performed genome walk proved the presence and expression of the formyltetrahydrofolate synthetase gene (FTHFS) under heterotrophic and syntrophic growth conditions [31]. Both SAO pathways require FTHFS activity, catalysing the reversible ATP-dependent activation of formate, and thus the encoding *fhs* gene is a suitable functional marker for both acetogenic and SAOB communities.

To date, assessment of acetogens has mainly been carried out using the FTHFSf/FTHFSr primer pair designed by Leaphart and Lovell [32]. These primers have successfully been used in diverse natural environments [32–39]. A few studies have also used these primers to examine potential acetogenic communities in anaerobic digesters [40–43]. However, only a low number of *fhs* sequences were recovered and with regard to syntrophic acetate oxidisers, the specificity of the FTHFSf/FTHFSr primer pair was limited, with only *C. ultunense* being detected [43]. The primer pair *fsh1*/FTHFSr, developed for quantification of the acetogenic community [44], is also restricted in targeting all known syntrophic acetate oxidisers [43]. The acetyl-CoA synthase gene, expressing the key enzyme of the carbonyl branch of the WL pathway, has also been used successfully as a functional marker for acetogenic community assemblages in bovine rumen and wallaby forestomach [45]. However, this gene is not suitable as a potential marker for the SAOB community, since only one of the suggested SAO pathways employs the carbonyl branch of the WL pathway.

Recently, Müller et al. [31] developed a degenerated *fhs* primer pair (3-SAO*fhs*), which successfully targeted the FTHFS genes of the known mesophilic SAOB and which was therefore used in the present study to extend knowledge of both the acetogenic community and the SAOB community, to follow the dynamic behaviour in anaerobic digesters, and to uncover potential new SAOB.

The study comprised a functional gene survey in anaerobic laboratory-scale and large-scale digesters targeting the FTHFS gene. First, the efficiency of the recently designed primer pair 3-SAO*fhs* [31] was evaluated by investigating the two laboratory-scale digesters previously studied using the FTHFSf/FTHFSr primer pair [43]. One of these digesters was operated at low and one at gradually increasing ammonia level, with the latter accompanied by a shift from aceticlastic to SAO-dominated methanogenesis [7]. The partial *fhs* genes amplified were analysed by terminal restriction fragment length polymorphism (T-RFLP) and clone-based comparisons. *fhs* genotypes correlating with increasing ammonia levels and SAO were identified and specific primers were designed in order to further evaluate their abundance and dynamics in other laboratory-scale and large-scale biogas processes operating at high ammonia levels with SAO as the predominant pathway for methane formation. In order to uncover and affiliate the potential SAOB community, the taxonomic distribution and dynamics of the bacterial community composition in the two laboratory-scale digesters were also investigated, by MiSeq Illumina sequencing targeting the 16S rRNA gene and by T-RFLP profiling.

## Results

### Acetogenic community composition and dynamics

#### Acetogenic community dynamic traced by *fhs*-TRFLP profiling

T-RFLP fingerprinting was conducted to follow the dynamics of the acetogenic community in digesters SAO1 and SAO3. SAO1 was operated with stable (control) and SAO3 with gradually increasing ammonia concentrations, which provoked a shift from aceticlastic methanogenesis to SAO after 225 days of operation in the latter [7].

**Fig. 1:**
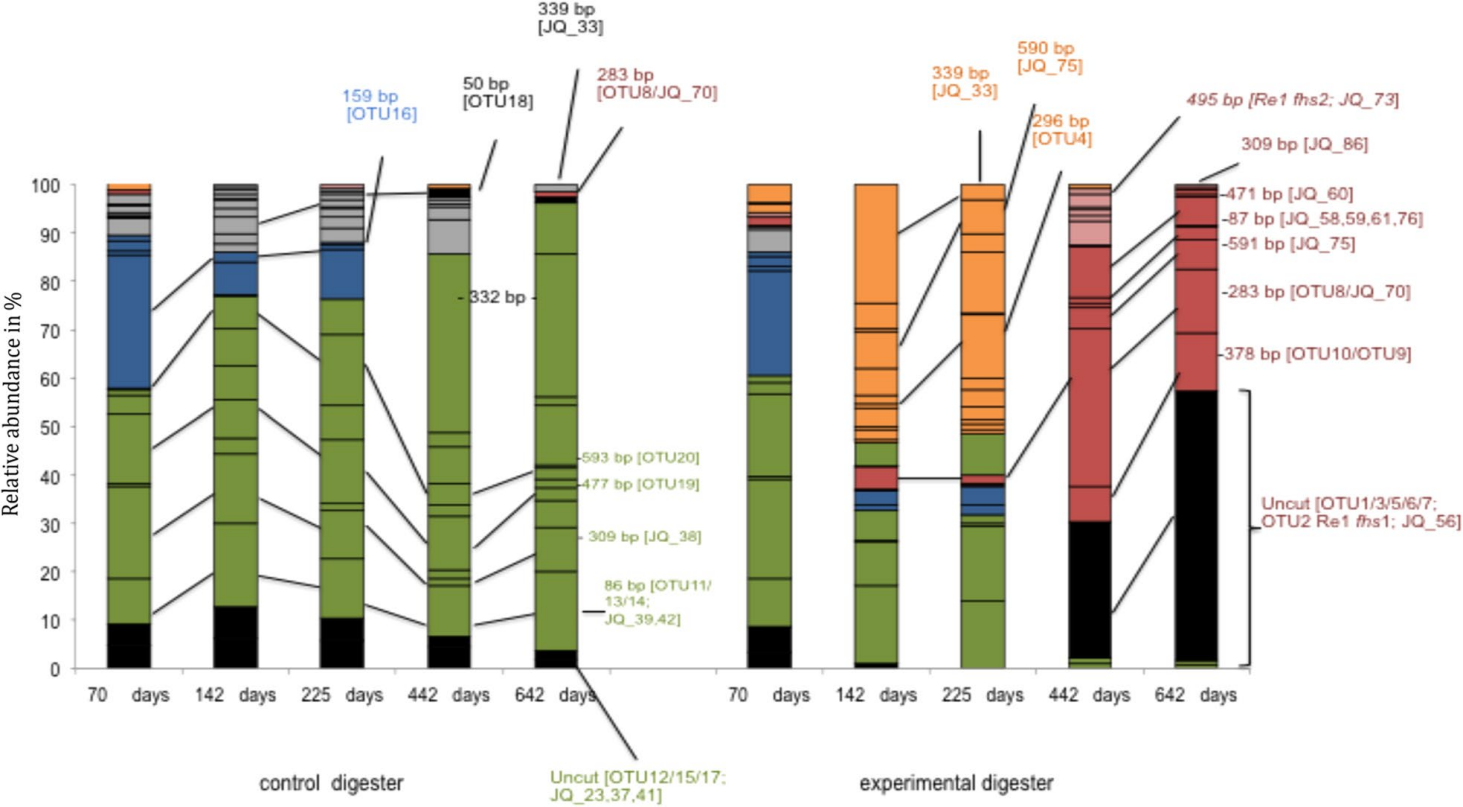
Dynamics of the acetogenic community in SAO1 (low-ammonia control digester) and SAO3 (high-ammonia experimental digester) traced by formyltetrahydrofolate synthetase (*fhs*) gene profiling using terminal restriction fragment length polymorphism (T-RFLP). Terminal restriction fragments (T-RFs) that could be affiliated to *fhs* genotypes are labelled by fragment size (bp) and operational taxonomic unit (OTU) affiliation or accession number. T-RFs were grouped into equally behaving fragments: Not establishing under either condition (*blue* 50, 159 bp), stable fragments in the control but fading in the experimental digester (*green* 86, 253, 238, 309, 477, 593 bp), establishing fragments up to 0.62 g NH_3_/L in the experimental digester, not detected in the control (*orange* 76, 220, 236/7, 240, 339, 442, 553, 590 bp), establishing fragments up to 0.96 g NH_3_/L in the experimental digester, not detected in the control (*red* 283, 379, 446, 471, 591 bp), exclusively detected at sample point day 442 in experimental digester (*pink* 60, 296, 463, 495/6, 520 bp). Fragments marked in *black* remained uncut. *Peaks* that emerged non-chronologically on one or two occasions are marked in *grey*. Days of operation are plotted on the *x*-axis, relative peak abundance on the *y*-axis

In SAO1, the *fhs* profile was relatively stable over the entire operating period of 642 days and was represented by the major terminal restriction fragments (T-RFs) 86, 238, 253, 309, 477 and 593 bp (Fig. 1, green bars) with two exceptions: T-RFs 239 and 332 bp emerged at the last two sampling points, at a relative abundance of more than 30 %, while another abundant T-RF with size 159 bp (Fig. 1, blue bar) declined during digester operation and was no longer detectable by day 442. The dominant T-RFs found in SAO1 were also present in the SAO3 digester on the first sampling occasion (day 70; 0.02 g NH_3_/L). However, on days 141 and 225, when the SAO3 digester was subjected to ammonia levels of around 0.09 and 0.30 g NH_3_/L, respectively (1.9 and 3.3 g NH_4_^+^-N/L, respectively), the T-RFLP profile indicated a distinct shift in the community, characterised by the major T-RFs 220, 236, 296, 339, 442, 553 and 590 bp (Fig. 1, orange bars). On days 442 and 630, at ammonia levels of around 0.62 and 0.96 g NH_3_/L (5.5 and 6.9 g NH_4_^+^-N/L), respectively, yet another community shift occurred whereby the previously observed T-RFs disappeared and instead the major T-RFs 87, 283, 379, 446, 471 and 591 bp became dominant (Fig. 1, red bars). A large proportion of the amplified partial *fhs* sequences remained uncut (Fig. 1, black bars). A few fragments were only observed on day 442 at an ammonia level of around 0.62 g NH_3_/L (Fig. 1, pink bars) including the T-RFs 60, 85, 296, 463, 495 and 520 bp.

#### Clone-based comparison of acetogenic community composition

A total of 79 and 61 clones recovered from SAO1 and SAO3 digesters, respectively, were sequenced. Sequences sharing 97–99 % nucleotide identity were considered an OTU; otherwise identical *fhs* sequences or sequences detected only once were considered to be “*fhs* clones” and named according their accession numbers (JQ0822…). The OTU and clone frequency (rank abundance) is presented in Additional file 1: Figure S1. In the experimental digester SAO3, 13 *fhs* clones and 10 *fhs*OTUs were recovered, designated OTU1-OTU10. Of the total partial *fhs* sequences recovered, 13 % were represented by OTU10, followed by OTU8, OTU7 and OTU5 (9.1 % each). For the control digester SAO1, 17 *fhs* clones and 10 *fhs*OTUs were obtained, designated OTU11-OTU20. Comparing the deduced amino acid sequences, OTU19 and OTU20 shared a pairwise identity of 98 %, representing together 27 % of the total partial *fhs* sequences recovered from SAO1. OTU11 and OTU14 had a pairwise identity of 92 % and represented in total 8 % of the *fhs* sequences recovered from SAO1. With the exception of OTU15 (4 % in SAO1), which was also recovered with one clone from the library of the experimental digester, none of the partial *fhs* sequences was recovered from SAO3 and vice versa. All OTUs found in the experimental and control digesters, as well as nine *fhs* clones in SAO3 and seven *fhs* clones in SAO1, were allocated to the T-RFLP pattern (Fig. 1). However, OTUs and clones were often represented by one and the same T-RF, as illustrated in Fig. 1. Moreover, the most highly abundant T-RF (332 bp) that emerged at the last two sampling points in the control digester could not be allocated to any of the clones obtained, and might thus be an enzymatic or technical artefact.

#### Abundance of ammonia-induced OTUs in other ammonia-stressed biogas processes

**Fig. 2:**
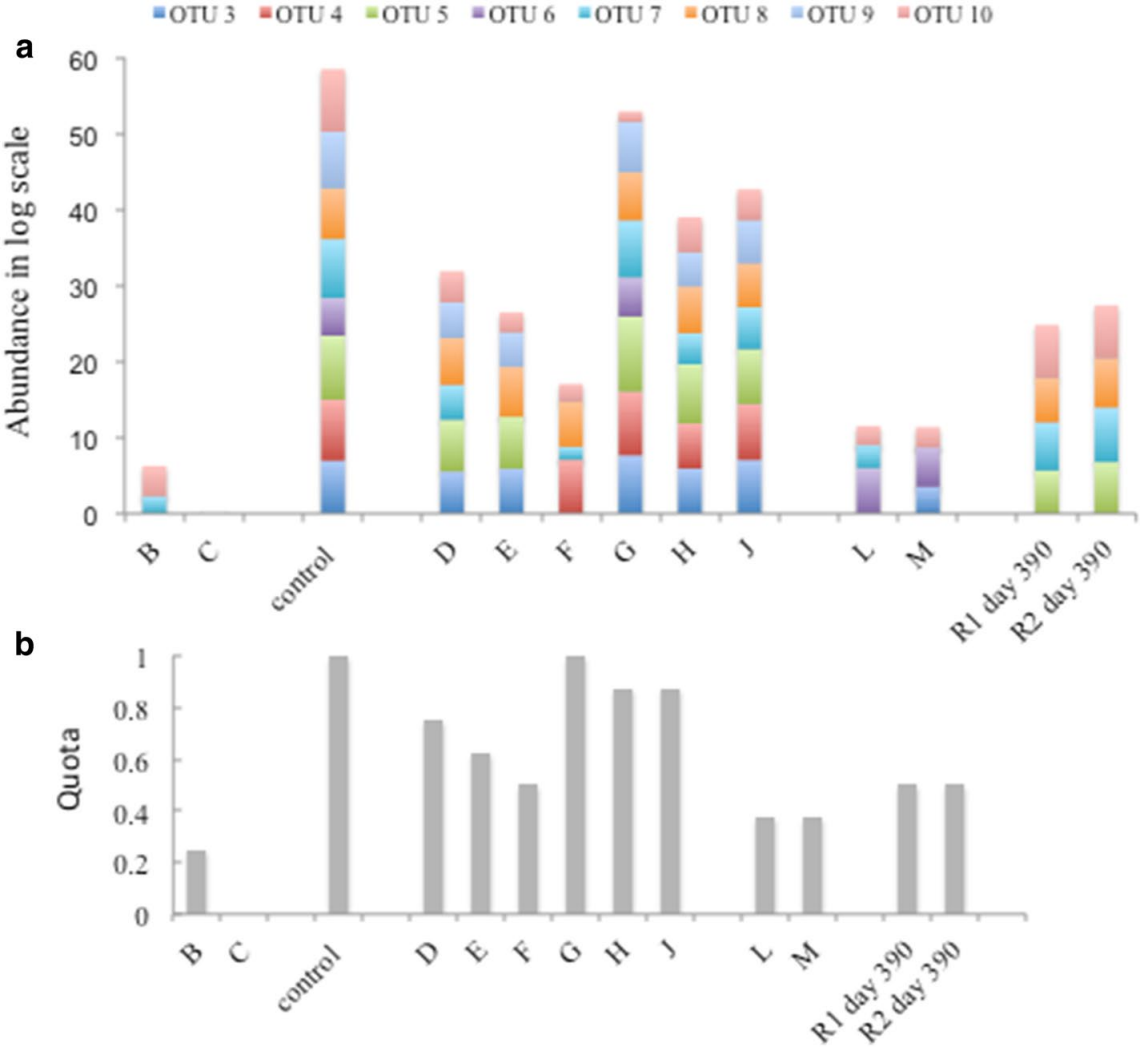
Abundance of operational taxonomic units (OTUs) 3–10 in industrial and laboratory-scale biogas processes, illustrated as: **a** log scale of gene copy numbers and **b** the ratio between number of OTUs present and total number of OTUs analysed. The presence of all OTUs gave a ratio = 1, as illustrated for the control (SAO3 day 442). Log scale values and standard deviation are summarised in Additional file 2: Table S1. Operation parameter and abundances of methanogens and known SAOB can be found in supplementary Additional file 9: Table S6 and Additional file 10: Table S7

Based on their numerical dominance in the ammonia-stressed SAO3 digester, OTUs 3–10 were selected as representatives for potential novel SAOB. OTU2 was excluded from further analyses since it appeared to be identical to the SAOB *T. acetatoxydans*. The abundance of this species has already been investigated in the digesters in question [12, 46]. In order to assess the occurrence of these selected OTUs, specific primers were designed for qPCR assays. The specificity of the primers and the qPCR efficiency are shown in Additional file 2: Table S1. The primers were used for investigation of ten large-scale biogas plants (B, C, D-H, J, L-M) and two parallel laboratory-scale digesters R1 and R2 (at day 390), operating at high ammonia level and dominated by SAO (Fig. 2a; Additional file 3: Table S2). The majority of the OTUs analysed were present in all SAO processes investigated. OTUs 3, 6, 7 and 10 were even found in the two thermophilic SAO digesters (L and M), but at lower abundance (Fig. 2a). In the low-ammonia, large-scale processes dominated by aceticlastic methanogenesis (B, C), only OTU10 and OTU7 were found to be present, and at significantly lower abundance than in the SAO-dominated processes. Relating the number of OTUs present to the total number of OTUs analysed, the highest ratio among the SAO-dominated processes was obtained for biogas plants G, H and J, and lower values for the thermophilic plants L and M. The lowest ratio was obtained for the aceticlastic processes (Fig. 2b).

**Fig. 3:**
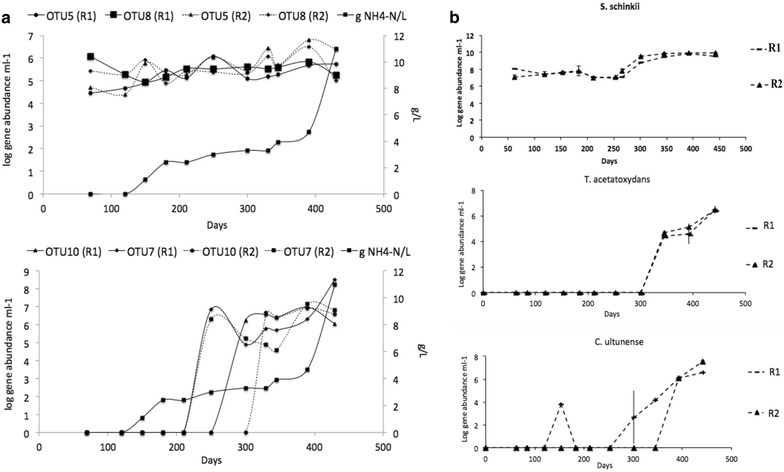
**a** Abundance of operational taxonomic units (OTUs) 5, 8, 7 and 10 in two parallel mesophilic laboratory-scale biogas digesters R1 and R2 subjected to increasing ammonia levels, illustrated as: log scale of *fhs* gene copy numbers obtained for OTU 5, 8 (*upper graph*), 7 and 10 (*lower graph*) and **b** average log gene abundance of the SAOB *S. schinkii* (*upper graph*) *T. acetatoxydans* (*middle graph*), and *C. ultunense* (*lower graph*) in R1 and R2 (*dashed lines*). **b** Re-drawn from data in Westerholm et al. [46]

The abundances of OTU3-10 were also traced over the entire operating period (430 days) in the two parallel laboratory-scale digesters R1 and R2 by analysing 11 sampling points. Among all OTUs investigated, OTUs 5, 7, 8 and 10 were found to be part of the microbial community (Fig. 3a). OTU5 and OTU8 were present from the beginning and increased slightly with increasing ammonia level, as observed for the SAOB *S. schinkii* (Fig. 3b) [46]. However, OTU7 and OTU10 were only detectable when the ammonium-nitrogen level exceeded 3 g/L and SAO became the dominant methane production process, a pattern previously reported for the SAOB *T. acetatoxydans* and *C. ultunense* (Fig. 3b) [46].

Considering all mesophilic, ammonia-rich samples (digesters D-H and J, SAO3 day 442, R1 day 390, R2 day 390), the OTUs 5, 7, 8 and 10 represented the most frequently found *fhs* genotypes (Additional file 4: Figure S2).

#### Phylogenetic affiliation of the recovered partial *fhs* sequences

**Fig. 4:**
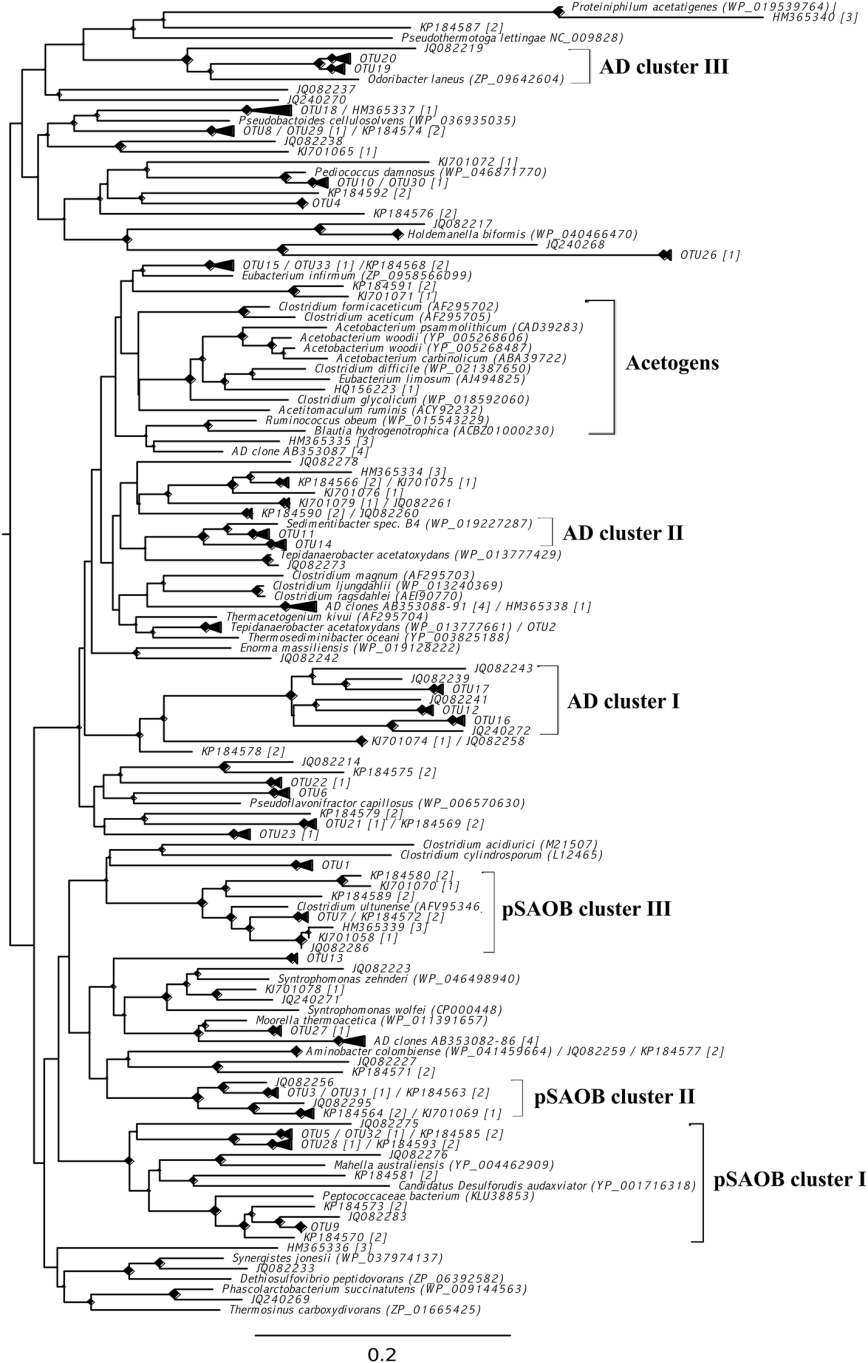
Phylogenetic placement in the maximum likelihood tree of the deduced formyltetrahydrofolate synthetase (FTHFS) amino acid sequences. Reference strains are given together with their accession numbers. *AD* anaerobic digestion, *pSAOB* potential syntrophic acetate-oxidising bacteria. The sulphate reducers formed a separate clade including the SAOB *S. schinkii* and *T. phaeum* as shown by Müller et al. [31] and were therefore excluded from the alignment. The tree was then re-build in order to reduce the size. (1) Westerholm et al. [48], (2) Moestedt et al. [47], (3) Westerholm et al. [43], (4) Hori et al. [40]

The deduced amino acid sequences of the recovered partial *fhs* sequences were phylogenetically analysed in relation to known acetogens, the current closest relative if sharing at least 80 % identity (Additional file 5: Table S3), sulphate reducers, known SAOB (Additional file 6: Table S4) and *fhs* sequences obtained from other biogas processes [40, 47, 48]. The partial *fhs* clones recovered from the low-ammonia control digester SAO1 formed three distinct clusters, designated AD (Anaerobic Digestion) clusters I-III (Fig. 4). The largest AD cluster, cluster I, is primarily represented by OTU12, OTU16 and OTU17. The closest relatives identified belong mainly to the families Thermoanaerobacteraceae and Ruminococcaceae (Additional file 5: Table S3). AD cluster II consists of OTU11 and OTU14, both sharing more than 90 % identity with a *Sedimentibacter* species, order Clostridiales (Additional file 5: Table S3). AD cluster III is mainly formed by OTU19 and OTU20, which cluster together with a member of the family Porphyromonadaceae as closest relative (Additional file 5: Table S3). OTU13, OTU15 and OTU18 were found dispersed in the tree and did not belong to any of the clusters identified.

For the ammonia-rich experimental digester SAO3, another three distinct clusters, designated potential Syntrophic Acetate-Oxidising Bacteria (pSAOB) clusters I–III, were found (Fig. 4). In general, these clusters appear distantly related to those found in the control digester SAO1. OUT9 and OTU5 form pSAOB cluster I, together with a bunch of *fhs* clones retrieved from SAO3 and other ammonia-rich processes [47, 48]. The current closest relatives identified affiliate to Thermoanaerobacterales families and Peptococcaceae (Additional file 5: Table S3). pSAOB cluster II encompasses OTU3 and three more *fhs* genotypes recovered from SAO3. The closest relatives found belong to the families Thermoanaerobacteraceae and Ruminococcaceae, but with identities below 80 % (Additional file 5: Table S3). Another bunch of partial *fhs* sequences including OTU7 shared up to 90 % identity with the SAOB *C. ultunense*, family Clostridiaceae (Additional file 6: Table S4), which together form the pSAOB cluster III. OTU1, OTU4, OTU6, OTU8 and OTU10 were found scattered throughout the tree. OTU10 shares high identity (96 %) with the FTHFS of *Pediococcus damnosus*, family Lactobacillaceae, a lactic acid producing bacterium commonly found in beer production [49] (Additional file 5: Table S3). OTU4, OTU8 and OTU6 are only distantly related to members of the Enterococcaceae, Ruminococcaceae and Clostridiales, respectively (Additional file 5: Table S3). OTU1 appeared to be distantly related to any reference strain included in the alignment. With the exception of OTU2 (97 % nucleotide identity to *T. acetatoxydans*) and AD cluster II, none of the OTUs or clones recovered branched together with characterised acetogens.

### Dynamic and taxonomic distribution of the bacterial community

MiSeq Illumina sequencing and T-RFLP profiling targeting the 16 s rRNA gene pool were used to investigate the whole bacterial community composition and dynamics in order to shed light on the phylogenetic affiliation of potential SAOB candidates, as indicated by the acetogenic community composition. The numbers of OTUs, coverage, Chao1, Shannon index and Simpson index values obtained per sample are summarised in Table 1.

**Table 1:** Summary of observed OTUs, Chao1, Shannon and Simpson index in digester SAO1 and SAO3

Sample	Sampling day	Chao1	Observed OTUs	Coverage (%)	Shannon	Simpson
SAO1.0	70	2225	2086	94	6.790	0.956
SAO1.2	141	2204	2049	93	6.402	0.937
SAO1.3	225	2076	1847	89	5.813	0.899
SAO1.5	442	2207	2063	93	7.174	0.970
SAO1.7	642	2413	2181	90	7.457	0.981
SAO3.0	70	2270	2047	90	6.481	0.939
SAO3.2	141	2022	1769	87	6.198	0.946
SAO3.3	225	1938	1614	83	5.684	0.912
SAO3.5	442	1281	1107	86	6.151	0.950
SAO3.7	642	851	763	90	5.256	0.907

**Fig. 5:**
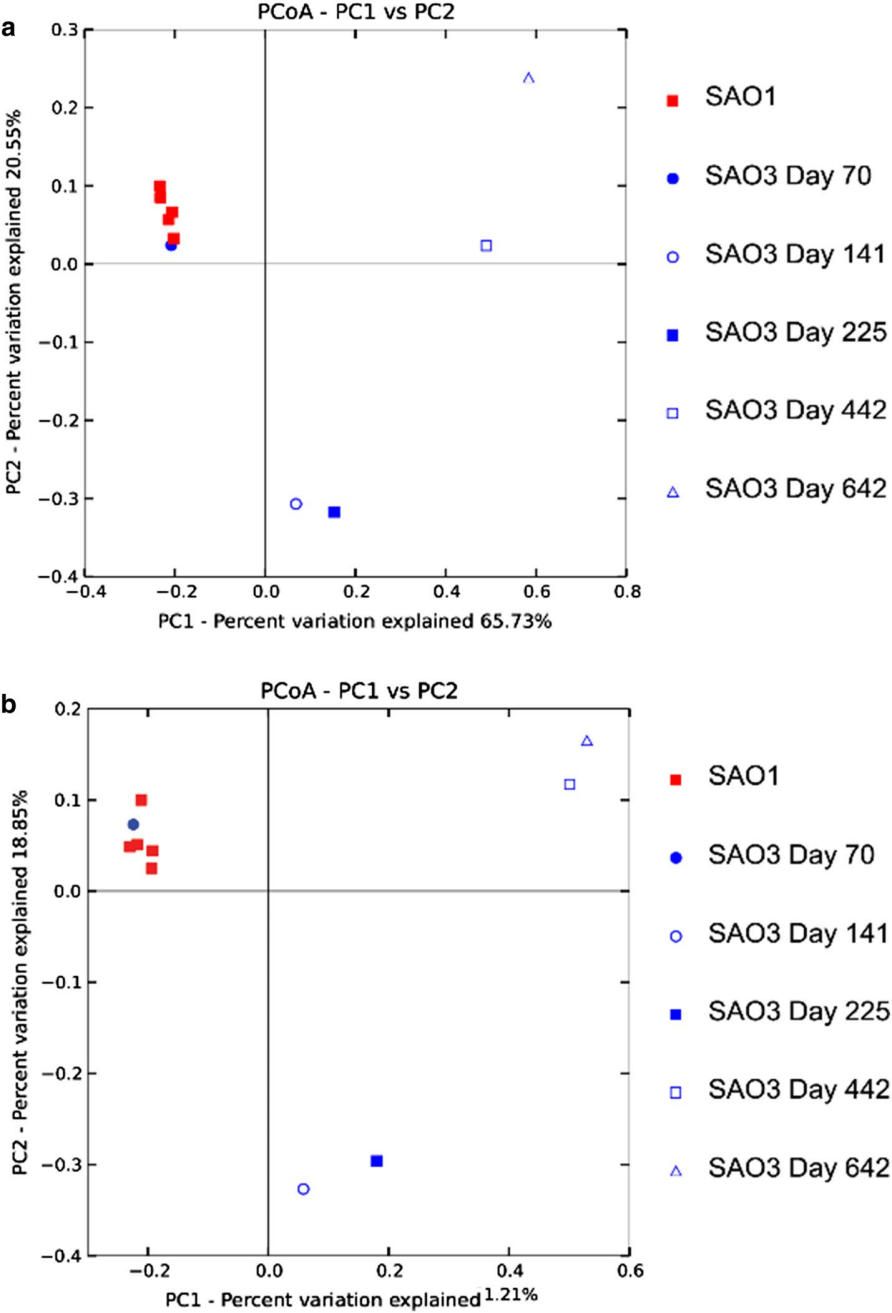
Principal coordinates analysis (PCoA) plot showing phylogenetic distances between SAO digester samples as determined by **a** unweighted UniFrac principal coordinate analysis and **b** weighted UniFrac principal coordinate analysis. *Red* SAO1; *blue* SAO3

The rarefaction analysis is displayed in supplementary Additional file 7: Figure S3. The estimated coverage indicated that, in the case of the control digester SAO1, the observed OTUs covered between 89 and 94 % of the bacterial community and in the case of the experimental digester SAO3 83–90 %. The number of OTUs in the experimental digester SAO3 decreased concurrently with rising ammonia level, whereas no similar trend was observed in the control SAO1. The Simpson index indicated slightly lower evenness with increasing ammonia level, with the lowest value (0.907) at sampling point day 642. The community in the control digester remained more even (0.981) at that sampling point. The Shannon index, accounting for both species abundance and evenness, revealed a similar trend, with the lowest value at day 642 in SAO3 (5.26) compared with the control at the same sampling point (7.46). Both weighted and unweighted UniFrac matrices PCoA plots revealed that all five SAO1 sampling points and the first SAO3 sampling point grouped close to each other, indicating similar phylogenetic composition. However, a significant phylogenetic distance was observed between the samples withdrawn from the control digester SAO1 and the samples from the experimental digester SAO3 subjected to increasing ammonia levels (Fig. 5).

**Fig. 6:**
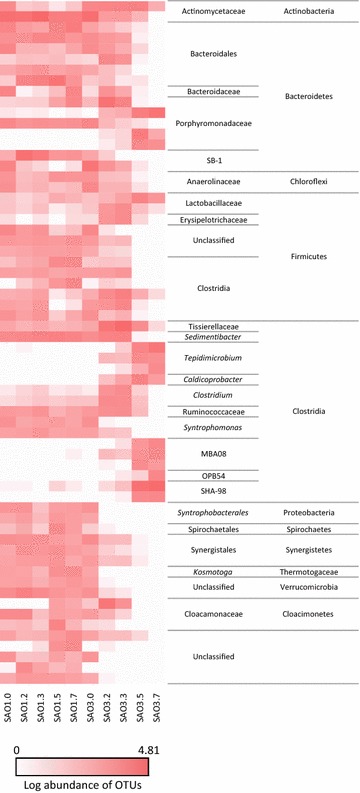
OTU heatmap based on bacterial OTUs having relative abundance higher or equal to 2.5 % with the process SAO1 and SAO3

On average, taxonomic assignment using the Ribosomal Database Project (RDP) classifier encompassed 85–97 % of the partial 16S rRNA gene sequences on phylum level and 45–77 % on family level. The bacterial community in the control digester SAO1 consisted of roughly equal proportions of the phyla Firmicutes (18–24 %), Bacteroidetes (23–31 %) and Actinobacteria (17–31 %). However, the latter dropped to 7 % after day 225 and was mainly replaced by Bacteroidetes (Fig. 6; Additional file 8: Table S5). The phylum Actinobacteria was strongly dominated by the family Actinomycetaceae (7–30 %). Within the phylum Bacteroidetes, unclassified Bacteroidales were most frequent (16–27 %), together with a smaller proportion of Porphyromonadaceae (2–7 %, Fig. 6). The highest diversity was observed within the phylum Firmicutes, in which most of the families belonged to the order Clostridiales, including Clostridiaceae (1.0–1.3 %), Syntrophomonadaceae (2–3 %), genus *Syntrophomonas*, Tissierellaceae (3–6 %), genus *Sedimentibacter*, and 1–3 % unclassified Clostridiales members. Members of the phyla Chloroflexi (2–5 %), Synergistetes (3–6 %), Verrucomicrobia (2–8 %) Proteobacteria (1–4 %), Planctomycetes (0.8–1.4 %), Spirochaetes (0.5–3 %), Tenericutes (0.4–1.5 %), Thermotogae (0.5–2.2 %) and WWE1 (0.2–6 %) were also present, but in smaller proportions. These minor phyla were mainly dominated by the families Anaerolinaceae (1–3.5 %), Syntrophaceae (0.2–2 %), including the genus *Syntrophus* (0.1–2 %), Thermotogaceae (0.1–2 %), including the genus *Kosmotoga* (0.4–1.7), Dethiosulfovibrionaceae (2–4 %), Cloacamonaceae (0.1–6 %), unclassified Bacilli (2–5 %) and 1–6 % unclassified Verrucomicrobia.

The bacterial community in the experimental digester SAO3 encompassed the same phyla as observed in the control digester at the first sampling point, but with increasing ammonia levels the proportions were significantly affected (Fig. 6; Additional file 8: Table S5). In particular, OTUs affiliated to the phylum Firmicutes increased from an initial relative abundance of 21 % up to 80 % between day 442 and day 642.

Conversely, the abundance of Bacteroidetes declined from 25% to 15–17 % at the last two sampling points. The phylum Actinobacteria was already strongly affected by the first ammonium increase at sampling point day 141, representing only 5 % of the bacterial community.

On family level, the impact of elevated ammonia levels on community composition was even more significant (Fig. 6; Additional file 8: Table S5) and can be summarised as follows: The gradual increase in ammonia level was accompanied by two distinct changes in the community composition. The first change occurred up to an ammonium level of 0.3 gNH_3_-N/L at sampling point day 225 and a second change in community composition occurred between day 225 and day 442, when ammonia level was further increased. The majority of the families observed in SAO1 declined in the experimental digester SAO3 with increasing ammonia levels and were completely replaced at the last two sampling points by other families or another genus belonging to the same family.

**Fig. 7:**
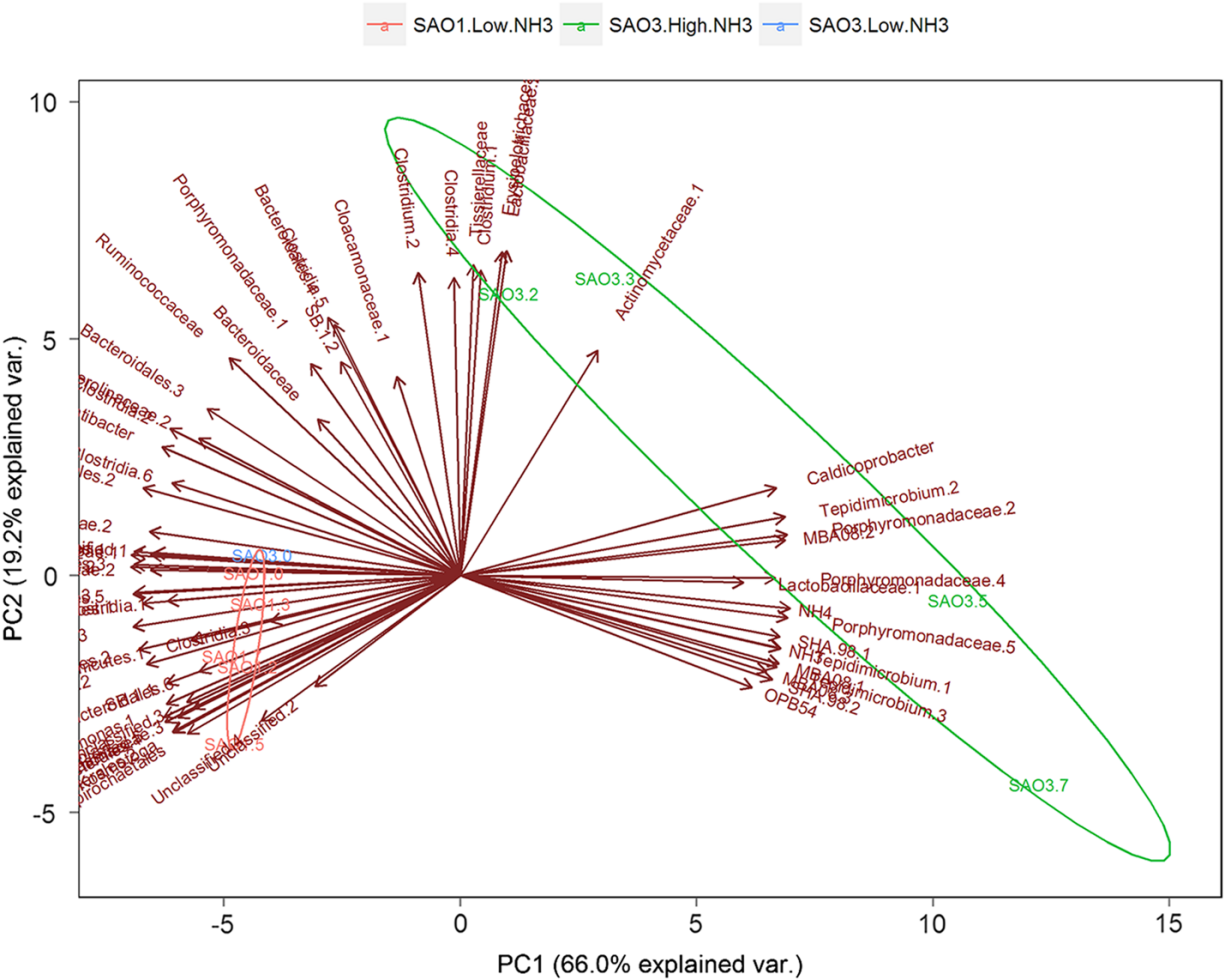
Correlation between bacterial community and the level of ammonium and free ammonia (g/L) within the process illustrated by using principal component analysis (PCA). The dominant bacterial operational taxonomic units (OTUs) that have relative abundance higher or equal to 2.5 % were selected as representative for bacterial community. All variables were centred and scaled before analysis. Results of the first two PCs were present within biplot. Sampling points day 70 (.0), day 141 (.2), day 225 (.3), day 442 (.5), day 642 (.7)

The phylum Actinobacteria was mainly represented by the family Actinomycetaceae (genus *Actinomyces*, 3–5 %). Similar low diversity on this taxonomic level was found for the phylum Bacteroidetes, which was represented only by members of the family Porphyromonadaceae, but at a significantly higher abundance (15–17 %) than in the control digester (1.6–6 %), at sampling points day 442 and day 642. The highest diversity and the most pronounced community change were observed within the phylum Firmicutes (Fig. 6; Additional file 8: Table S5). The first community change in response to increasing ammonium levels was characterised by a rise of Clostridiaceae, dominated by genus *Clostridium* (up to 7 %), Eubacteriaceae, genus *Eubacterium* (up to 1.3 %), Ruminococcaceae (up to 2.5 %), Acidaminobacteraceae, genus *Guggenheimella* (up to 1 %), Tissierellaceae, unknown genus (up to 31 %), Erysipelotrichaceae (up to 2.3 %), Lactobacillaceae (up to 2.3 %) and Leuconostocaceae, genus *Leuconostoc* (up to 1.2 %), as well as up to 12 % unclassified Clostridiales members. However, members of the family Cloacamonaceae (up to 12 %) belonging to the WWE1 candidate division also increased. The remaining changes were an increase of <0.4 %, at family level (Additional file 8: Table S5). However, once ammonia concentration exceeded 0.30 g NH_3_/L, this bacterial community was replaced by members of the following taxa: Clostridia order SHA-98 (up to 31 %), and MBA08 (up to 8 %), Tissierellaceae, genus *Tepidmicrobium* (up to 19 %), Caldicoprobacteraceae, genus *Caldicobacter* (up to 5.0 %), Clostridiaceae, mainly genus *Alkaliphilus* (up to 1.8 %), Clostridiales (0.5–4.0 %), Thermoanaerobacterales (1.1–1.4 %), Thermoanaerobacteraceae, genus *Thermacetogenium* (up to 0.9 %), and unclassified Firmicutes (up to 0.9 %), Enterococcaceae (up to 1.7 %), Lactobacillaceae, unknown genus (up to 6 %) and Acholeplasmataceae (up to 0.6 %), the latter belonging to the phylum Tenericutes (Figs. 6, 7; Additional file 8: Table S5). All other changes observed were below 0.1 %.

A similar community dynamic was observed using T-RFLP analysis (Additional file 7: Figure S4). A cluster of bacteria represented by T-RFs labelled orange increased until the ammonia concentration reached 0.30 g/L at day 225, but declined at higher ammonia levels. Instead, another group of bacteria labelled red in the T-RFLP profile emerged and replaced the bacterial community dominating under low-ammonia conditions (labelled blue, Additional file 7: Figure S4).

## Discussion

### Coverage evaluation of the 3SAO*fhs* primer pair and acetogenic assemblage

The 3SAO-*fhs* primers used in the present study clearly generated more diverse *fhs* profiles, including number of T-RF peaks and *fhs* clones, than the FTHFSr/FTHFSf primer pair used previously by Westerholm et al. [43]. The dynamic trend indicating two community shifts in the high-ammonia experimental digester between days 141–225 and after day 442, as also observed in the study by Westerholm et al. [43], was more distinct with the 3SAO*fhs* primers. In total, 59 unique OTUs/clones were recovered from the two digesters (Additional file 1: Figure S1; Additional file 5: Table S3), compared with the eight clones recovered in the study by Westerholm et al. [43], while three were recovered by both primer pairs. The partial *fhs* clone HM365339, which Westerholm et al. [43] found to be most abundant in the experimental SAO3 digester, was low in abundance here (JQ82286). The corresponding T-RF emerged in the present T-RFLP profile at the last sampling point, but with only 1/100 (0.84 %) relative abundance, compared with 80 % abundance obtained in the previous study using the FTHFS primer set [43]. Thus, the FTHFSr/FTHFSf primer pair targeted only a part of the acetogenic community and enriched less abundant genotypes.

In this context, it should be noted that many species harbour two *fhs* genes. In the case of the known SAOB, this allelic genotype has been demonstrated for *C. ultunense* and *T. acetatoxydans* [31] and for the thermophilic SAOB *T. phaeum* [27]. Thus, discrepancies between observed and actual species richness most likely occur during *fhs* profiling.

The 3SAO*fhs* primer pair was successfully shown to target the phylogenetically distantly related SAOB *T. acetatoxydans*, *C. ultunense* and *S. schinkii* [31]. In previous quantitative studies using species-specific primers targeting 16S ribosomal RNA genes, these three known SAOB were all present in the experimental digester SAO3, but were not recovered using the FTHFSr/FTHFSf primers [43, 46]. The clone libraries constructed in the present study recovered the two partial *fhs* genes of *T. acetatoxydans*, where *fhs*1 was represented by OTU2 and *fhs*2 by clone JQ82273. However, the corresponding T-RF 495 bp was present in very low abundance and emerged exclusively at sampling point day 442 (Fig. 1, pink bars). The corresponding *fhs* sequences for *C. ultunense* and *S. schinkii* were not recovered in the present study. However, this result is reasonable considering that according to qPCR results, these bacteria are reported to be equally or even less abundant than *T. acetatoxydans* [46].

The partial *fhs* sequences obtained in this study were mainly found not to group with characterised acetogens (Fig. 4), as has also been reported for the majority of the *fhs* genes of the known SAOB [31]. The *fhs* genes of the SAOB *S. schinkii* and *T. phaeum* are clearly distinguished from acetogenic *fhs* genes and are instead more closely related to those of sulphate reducers [31]. Two previous studies employing the FTHFSr/FTHFSf primer pair for investigation of acetogenic communities in biogas reactors [40, 42] also recovered only a limited number of partial *fhs* sequences. Thus, the acetogenic assemblages in anaerobic digesters differ clearly from the acetogenic communities in other anaerobic environments, which have been successfully revealed by the FTHFSr/FTHFSf primer pair [33–39, 44, 50–53].

It is worth noting in this regard that the isolated mesophilic SAOB differ metabolically from many homoacetogens by their lack of ability to perform autotrophic acetogenesis [22–24, 29].

### Potential novel syntrophic acetate-oxidising bacteria

All three approaches (Illumina sequencing, clone-based comparisons and T-RFLP analysis) revealed a significant impact of increasing ammonia level on the bacterial community composition. Both the entire bacterial community and the *fhs*-harbouring sub-community responded by a complete community change, as illustrated in Figs. 1, 5, 6, 7, Additional file 1: Figure S1, Additional file 7: Figure S4 and Additional file 8: Table S5. Comparing the phylogenetic composition, a pronounced shift on family level was already observed in response to elevated ammonia up to a concentration of 0.3 g/L. The further gradual increases in the ammonia concentration in this study up to 0.96 g/L (225 days of operation) caused a second community shift finally resulting in a complete change in the community. This result contradicts findings by Werner et al. [54], who did not detect any marked impact of elevated ammonia concentration on the microbial community when performing a similar study by increasing the ammonia level up to 0.2 g/L by adding ammonium chloride. Thus, rather than representing a shift to SAO, the first community shift observed in the present study was most likely correlated with the increase in albumin, gradually added in order to increase the free ammonia level. This gradual increment in proteinaceous material was most likely the cause of the enrichment of OTUs affiliating to Porphyromonadaceae, Actinomytaceae, Lactobacillaceae and Caldicoprobacteraceae. The abundances of these families increased gradually in the experimental digester over time and, but were at very low levels in the control digester, where the feedstock did not change over time. Representatives of the family Porphyromonadaceae have recently been isolated from mesophilic biogas reactors and have been described as specialising in proteolytic degradation [55, 56]. Albumin-enhanced growth has also been reported for members of the genus *Actinomyces* [57]. Members of the genus *Caldicoprobacter* have been described as thermophilic, xylanolytic bacteria that can be enriched on proteinaceous substrates together with sugar and sugar derivatives [58, 59]. Lactobacillaceae are known to be specialists in degradation of polysaccharides [60] and have previously been observed to be enriched in response to glucose addition in batch digesters [61] and during two-stage digestion of maize silage [62]. However, a recent publication suggested that representatives from this family are indirectly engaged in protein degradation by inducing the expression of peptidase genes in the gut of Drosphila [63].

In contrast, OTUs affiliating to *Tepidimicrobium* (Tessierellaceae), *Thermacetogenium* (Thermoanaerobacteraceae), *Alkaliphilus* (Clostridiaceae), Acholeplasmataceae, MBA08 (Clostridia), SHA-98 (Clostridia), OPB54 (Firmicutes) and Thermanaerobacterales did not correlate to increasing albumin levels, since they became more abundant first at the same time point as SAO became the dominant methane producing pathway, i.e. at the last two sampling points, on day 442 and day 642. Thermoanaerobacterales, Thermoanaerobacteraceae and Clostridiaceae contain the characterised SAOBs *T. phaeum,T. acetatoxydans*, *S. schinkii* and *C. ultunense*. The latter three have also been shown to have similar dynamics and are reported to be present in very low numbers below ammonia levels of 0.3 g/L [7, 46].

Similarly to the rise of these OTUs and known SAOBs, the *fhs* T-RFLP profile obtained in this study encompassed a bunch of fragments (displayed in light pink and red in Fig. 1) following the same dynamic, i.e. an increase on day 442. The corresponding *fhs*OTUs and clones recovered from the high-ammonia conditions between 0.62 and 0.96 g/L were also found clustering together with representatives of the Thermoanaerobacterales (pSAOB cluster I), Thermoanaerobacteraceae (pSAOB cluster II) and Clostridiaceae (pSAOB cluster III), and the majority of the *fhs*OTUs were also detected in other high-ammonia biogas processes as investigated here and by Moestedt et al. [47].

Thus, taxa emerging at the last two sampling points most likely include potential SAOB. Members of Clostridiales, Thermoanaerobacteraceae and Acholeplasmataceae have also been identified in previous studies as correlating with SAO activity [54, 64]. However, we did not observe any correlations between SAO and the presence of Spirochaetes or Thermotogae members as reported by Lee et al. [65] and Nobu et al. [30].

As observed by both molecular approaches performed, the increase in protein/ammonia induced a complete community shift, which encompassed the bacterial community on the whole. Thus, SAO capability appears to affiliate to populations not detectable under low-ammonia conditions, and which are specialised to that particular niche rather than possessing metabolic versatility. This agrees with findings of cultivation experiments and genome-scale analysis confirming restricted metabolic capacities in the case of the mesophilic SAOB *C. ultunense,T. acetatoxydans* and *S. schinkii* [22–24, 29, (Manzoor et al., unplublished)] and low abundance of *T. acetatoxydans* and *C. ultunense* in biogas processes operating at low-ammonia levels [9, 12, 46]. However, when elevated ammonia levels repress the competing aceticlastic methanogens, these ammonia-tolerant specialists extend and increase in abundance, as has been described for *T. acetatoxydans* and *C. ultunense* [9, 46] and as was observed in this study for *fhs*OTU7 and *fhs*OTU10. Although metabolically most restricted, *S. schinkii* has also been found at relatively high abundance in low-ammonia processes, suggesting that this species has stronger competitive ability [9, 12, 46]. Likewise, *fhs*OTU5 and OTU8, recovered in this study, showed similar dynamic behaviour as observed for *S. schinkii*. Recent genome-scale and transcriptomic analyses have identified traits most likely conveying competiveness in the case of *S. schinkii* under low-ammonia conditions (Manzoor et al., unpublished). The genome of *T. acetatoxydans* did not reveal such attributes [29].

## Conclusions

Current understanding of the SAO pathway and of the organisms occupying this niche is restricted due to the limited number of SAOB available in pure culture and the lack of molecular tools for identifying and studying the diversity and distribution of bacteria potentially performing SAO. In this study, different techniques (T-RFLP, quantitative PCR, clone-based analyses deep sequencing approach) and targets (*fhs* and 16S rRNA genes) were combined and the results revealed a potential SAO community characterised by low abundances. The results of this study point to a keystone community specialised in SAO rather than a versatile, heterogeneous community performing SAO as a metabolic option. The phylogenetic affiliation and population dynamics observed using different techniques and targets indicate that members of the Clostridia class, mainly belonging to the orders Clostridiales and Thermoanaerobacterales, primarily perform SAO under mesophilic conditions.

Although the molecular tools used here did not allow direct discrimination of species providing SAO capabilities, a frequent presence in SAO-dominated processes, as illustrated by qPCR and phylogenetic affiliations, indicates potential involvement. Overall, the present results provide evidence of the functional importance of the bacterial families identified in high-ammonia systems and extend existing knowledge of bacterial and acetogenic assemblages at low and high ammonia levels. This information is valuable when monitoring and assessing impacts on the syntrophic acetate-oxidising communities and when identifying the characteristics of robust, productive biogas processes relying on SAO.

## Methods

### Digester samples

Samples used for the microbial analysis were taken from several previously investigated reactors, and operational data from these are available in previously published work [7, 9, 12] and are summarised in supplementary Additional file 10: Table S7. In these digesters, the abundance of different methanogens and the known SAOB has also been analysed previously (Additional file 9: Table S6) [9, 12, 46]. The digesters included in this study were four laboratory-scale digesters (SAO1, SAO3, R1, R2) and 10 large-scale biogas processes (B-H, J, L-M) (Additional file 9: Table S6, Additional file 10: Table S7). In short, SAO1 and SAO3 operated at mesophilic temperature conditions (37 °C) for 642 days and were fed with municipal household waste. SAO3 was subjected to an decrease and increased level of household waste and egg albumin, respectively, resulting in increasing ammonia/ammonium levels over time (0.8–6.9 g NH_4_^+^-N/L, 0.02–0.96 g NH_3_/L) and a shift from aceticlastic methanogenesis to SAO between 225 and 442 days of operation. SAO1 operated as an aceticlastic ‘control’ digester at low-ammonia levels (0.65–0.90 g NH_4_^+^-N/L, 0.01–0.03 g NH_3_/L) over the whole experimental period and was sampled concurrently [7, 46]. R1 and R2 were two mesophilic (37 °C) laboratory-scale digesters fed a mixture of cattle manure and whole stillage. The digesters were exposed to gradually increasing ammonia levels over time by enhanced organic loading rate and replacement of whole stillage with egg albumin (R1: 0.07–0.43 NH_3_ g/L; R2: 1.5–11 g NH_4_/L), which induced a shift from aceticlastic methanogenesis to SAO. The 10 large-scale biogas processes (B-H, J, L-M) that were sampled and included in the survey operated under the following conditions: B-C: mesophilic low ammonia (<0.1 g NH_3_/L, non-syntrophic); D-H: J mesophilic high ammonia (0.1–0.8 g NH_3_/L); and L-M: thermophilic high-ammonia (>0.6 g NH_3_/L), with SAO as the dominant pathway for methanogenesis (Additional file 9: Table S6, Additional file 10: Table S7) [12]. All samples were stored at −20 °C prior to analysis.

### Extraction of genomic DNA

Triplicate samples of genomic DNA were extracted from each 0.3 mL digester sample using the FastDNA soil kit from MP Biomedicals (France) and the MP Biomedicals homogeniser (speed 5.5 for 40 s) according to the manufacturer’ instructions. No replicates were prepared for R1 and R2, since these digesters were operated in parallel under similar conditions.

### Polymerase chain reaction (PCR) techniques

The touchdown PCR procedure described by Müller et al. [31] was performed before both *fhs* T-RFLP analysis and *fhs* clone library construction, but the annealing temperature was increased to 72 °C and the cycle number reduced to 28. The reaction system consisted of 35 ng genomic DNA and 20 pmol of each 3-SAO*fhs* primer [31]. To reduce background noise, the respective band at approx. 630 bp was gel-purified using the QIAquick Gel Extraction Kit (Qiagen, Hilden, Germany). PCR amplification of bacterial 16S rRNA gene for T-RFLP analysis was carried out as described by Leven et al. [66]. For T-RFLP analysis, the respective forward primers were labelled with 6-carboxyfluorescein (FAM). DNA was visualised by gel electrophoresis (1.5 % agarose in 0.5× Tris-borate-EDTA buffer).

Operational taxonomic unit (*fhs*OTU) primer specificity (Table 2) was confirmed by standard PCR using selected industrial DNA samples, iQ PCR Supermix (Biorad, Hercules, CA USA) and 10 pmol/μL of each primer. Amplification was achieved by 95 °C for 5 min, 35 cycles at 95 °C for 30 s, annealing according to Table 2 for 60 s, 72 °C for 30 s and final elongation at 72 °C for 10 min. PCR products were purified by either QIAquick Gel Extraction or QIAquick PCR purification Kit (Qiagen) prior to Sanger sequencing. Sanger sequencing was performed by Uppsala Genome Center (Uppsala, Sweden). Primers produced in this study were designed using Geneious v5.4 and synthesised by Invitrogen (Thermo Fisher Scientific, Waltham, CA, USA).

**Table 2:** Primers used in quantitative, touch down and standard PCR targeting the *fhs* gene

Primer name	Nucleotide sequence (5′–3)	Annealing temperature (°C)
OTU3 fw OTU3 rev	TTTGAACCAGCTGGGGAAA TTAGCTCGCATTGTTGTCG	63
OTU4 fw OTU4 rev	GTTGAGGAAAATCGGRAAA CTATCCAATATTATCATTG	55
OTU5 fw OTU5 rev	CATACGTGAGCCTTCCCTGG CCGCAAACGATATTCCGCAG	63
OTU6 fw OTU6 rev	ATTACATAGCTTAGGCAAA CCACCACAATATTGCCAAG	53
OTU7 fw	AGAGAACCATCTTTAGGGCCA	63
OTU7 rev	TGGTTTTCCACCAAGACCCA	63
OTU8 fw OTU8 rev	GAGAATTGGCAAGAAGGCCA CTGCCTCCCAGACCAACTAC	63
OTU9 fw OTU9 rev	CCTGGGCAAGCTGGACAAG GTGGGCAAAATCTTGGTTG	63
OTU10 fw OTU10 rev	TTTGCGAAAATTAAATAAG ATTAACCGGATTGTCATTG	55

*fhs* clone libraries were analysed by colony PCR using 10 pmol/L M13 primer for each (fw: GTAAAACGACGGCCAGTG, rev: GGAAACAGCTATGACCATG, Thermo Fisher Scientific) and GE PuReTaq Ready-to-go PCR beads (GE Healthcare, Buckinghamshire, UK) applying the following PCR cycles: 95 °C for 10 min, 35 cycles of 95 °C for 1 min, 55 °C for 1 min, 72 °C for 45 s and 72 °C for 10 min. Purification and sequencing were conducted as described above.

Quantitative PCR (qPCR) was performed in triplicate (unless otherwise stated) using a BioRad iCycle (Hercules, CA). Each reaction contained 10 μL iQ SYBR green PCR Supermix (Biorad), 5 μL sterile de-ionised water, 1 μL (10 pmol/μL) of each OTU-specific primer (Table 2) and 3 μL DNA sample (diluted 1:10, 1:25 or 1:50). The DNA concentrations of undiluted samples were between 40 and 180 ng/mL. qPCR was conducted as follows: 95 °C for 7 min, 50 cycles at 95 °C for 30 s, annealing according to Table 2 for 60 s and elongation at 72 °C for 30 s. A temperature melt curve (55–95 °C, Δ*T* = 0.1 °C) was performed at the end of every PCR assay.

OTU standard curves were constructed by performing standard PCR as described above using total genomic DNA purified from experimental digester SAO3 at sampling point day 442. PCR products were purified and cloned using the pGEMTeasy vector system (Promega, Fitchburg, WI, USA) as recommended by the manufacturer. Chemically competent *Escherichia coli* JM109 cells (Promega) were transformed using the purified PCR products following the manufacturer’s instructions. Successful cloning was confirmed by colony PCR and sequencing as described above. Standard curves consisted of purified plasmid (Qiagen, Plasmid Purification Kit) diluted to 10^8^–10^0^ copy numbers.

### T-RFLP analysis

*fhs* gene T-RFLP data sets were collected as triplicates from each sampling point. PCR products were generated and purified as described above and digested with *Hpy*188III (Fermentas), as recommended by the manufacturer. All digestates were diluted 1:10 with sterile de-ionised water before analysis. Separation of T-RFs was done by Uppsala Genome Center (Sweden) using the MapMarker 1000 (Rox) size standard and an ABI3730XL DNA analyser (Applied Biosystems). The fragment data obtained were analysed with Peak Scanner v1.0 software (Applied Biosystems). The resulting profiles were further processed and evaluated in Excel (Windows, Micosoft Office Version 2007), with the threshold value set at 0.5 % of total peak abundance.

### Construction of clone libraries

*fhs* clone libraries were constructed from digester SAO1 at the sampling points days 225 and 442 and from digester SAO3 on days 442 and 642. Touchdown PCR was conducted as described above from triplicate DNA extractions. The approximately 630 bp products were purified, pooled and recovered in chemically competent *E. coli* JM109 cells as described above. 61 clones from the experimental digester and 79 clones from the control digester were analysed by colony PCR and sequencing. A total of 18 clones were found to be harbouring non-*fhs* fragments. The nucleotide sequence data reported in this study were deposited at GenBank with accession numbers JQ082213-JQ082254 for clones obtained from the control digester (SAO1) and JQ082255-JQ082297, JQ240268-JQ240272 for clones obtained from the experimental digester (SAO3).

### Primer design and phylogenetic analysis of partial *fhs*

*fhs*OTU primer pairs were designed using Geneious software version 5.4 (Biomatters, Auckland, New Zealand) and Primer3 version 0.4.0 [67]. Specificity was confirmed both in silico, using the BlastN search algorithm provided by the National Library of Medicine, and in vitro, by sequencing as described above (data summarised in Additional file 2: Table S1).

Sequence assembly, editing and manual chimera checks were conducted using Geneious software. Multiple sequence alignments were performed from the deduced amino acid sequence of partial *fhs* sequences, reference sequences and uncultured clones taken from the NCBI database (accession numbers are given in the tree) using MAFFT v7.017 [68]. A maximum likelihood tree was constructed using PhyML v3.0 based on 100 bootstraps [69]. Both MAFFT and PhyML were implemented in Geneious.

### Construction of 16S amplicon libraries for Illumina sequencing

16S rRNA amplicon libraries were constructed as triplicates by two successional PCR procedures. First, the 16S rRNA pool was amplified using 10 μM each of the primers 515`F and 805R [70], 5–10 ng DNA and the Phusion High-Fidelity PCR master mix from Thermo Scientific (Waltham, MA, USA) in a total volume of 25 μL. Amplification was achieved by the following PCR conditions: 98 °C for 30 s, 20 cycles of 98 °C for 10 s, 60 °C for 30 s and 72 °C for 4 s, followed by a final elongation step of 72 °C for 2 min. Successful amplification was visualised by gel electrophoresis (2 % agarose in 0.5× Tris-borate-EDTA buffer). PCR products were purified using magnetic beads (AMPure XP, Beckman Coulter Genomics, Beverly, MA, USA) according to the manufacturer’s protocol (beads/sample ratio 0.8). 20 μL deionised water was used as eluant. Illumina-compatible barcodes and adapters [70] were attached at both ends in the second amplification step using the following protocol: 98 °C for 30 s, 8 cycles of 98 °C for 10 s, 62 °C for 30 s and 72 °C for 5 s, followed by a final elongation step of 72 °C for 2 min. The reaction consisted of 10 μL cleaned PCR product, 10 μM each primer and Phusion High-Fidelity PCR master mix in a total volume of 25 μL. Before purifying as described above, the quality of PCR products was visualised using gel electrophoresis. The amplicon libraries were finally quantified using Qubit (Invitrogen, Thermo Fisher Science, Waltham, MA, USA) and subsequently pooled in equal molar amounts. Sequencing was performed at SciLifeLab Stockholm, Sweden using the MiSeq Illumina sequencing platform. The library was spiked with 15 % of Phix control to improve base calling during sequencing.

### Illumina sequencing data analysis

The paired end reads obtained were first trimmed with Cutadapt [71], using the following criteria: The adaptor and primer sequences were trimmed. Any base from the 3′ end which had a quality below 10 was trimmed. Any read was discarded if it contained N base, was longer than 300 bp, or did not contain primer sequences. The trimmed paired end reads were further processed in Quantitative Insights into Microbial Ecology (QIIME, 1.7.0/1.8.0) [72]. Join_paired_ends.py was used for joining paired end reads using the SeqPrep method (https://github.com/jstjohn/SeqPrep), with minimum overlap base 150 bp. The joined reads were then used for splitting into libraries, no barcode errors were allowed, and only one consecutive low-quality base call was allowed per read. Reads. Phred quality scores below 20 were discarded. Subsequently, the operational taxonomic units (OTUs) were assigned using the open reference OTU pick strategy [73]. The OTU clustering was set to a threshold of 97 % similarity and performed with Uclust [74] against Greengenes core set (gg_13_8). The most abundant sequence in each OTU was selected as a representative sequence and further aligned against the Greengenes core set using PyNAST software [75]. The chimeric sequences were removed by ChimeraSlayer [76]. Taxonomy was assigned to each OTU using the Ribosomal Database Project (RDP) classifier with a minimum confidence threshold of 80 % [77]. The alignment was filtered to remove gaps and hypervariable regions using a Lane mask, and a maximum likelihood tree was constructed from the filtered alignment using FastTree [78]. The final OTU table was filtered based on: One OTU had to be observed in three samples to be retained and one OTU had to contain 36 reads (0.001 % of total reads) to be retained. Alpha diversity measures included Chao1, Shannon and Simpson index and species richness. Beta diversity measures included unweighted and weighted UniFrac distance matrix and were computed and plotted using QIIME [72]. Sequences have been deposited in the NCBI Sequence Read Archive (SRA) under accession number SRP064504.

## Additional files

10.1186/s13068-016-0454-9 Rank abundance of formyltetrahydrofolate synthetase (*fhs*) operational taxonomic units (OTUs) and genotypes recovered in clone libraries constructed from: A) low-ammonia control digester SAO1 and B) high-ammonia experimental digester SAO3. Rank abundance (%) was calculated based on the total number of *fhs*-harbouring clones obtained. In silico *Hpy*188III restriction sites are given in brackets.
10.1186/s13068-016-0454-9 Summary of qPCR amplification efficiency and primer specificity. OTU primer specificity was confirmed by standard PCR using selected industrial DNA samples as listed in the table.
10.1186/s13068-016-0454-9 Summary of OTU gene copy numbers in log scale.
10.1186/s13068-016-0454-9 Operational taxonomic unit (OTU) frequency ratio comprising all mesophilic ammonia-rich samples analysed (a value of 1 indicates the presence of the respective OTU in every sample analysed). A total of nine samples were included (industrial biogas plants D-H, J; laboratory-scale digesters SAO3 day 442, R1 and R2 day 390).
10.1186/s13068-016-0454-9 Summary of the partial *fhs* sequences and OTUs obtained, the in silico T-RFLP fragment and the closest relative.
10.1186/s13068-016-0454-9 Distance-matrix of the deduced amino acid sequence of recovered partial *fhs* sequences and known SAOB constructed by MAFFT v7.017.
10.1186/s13068-016-0454-9 Dynamics of the bacterial community in SAO1 (low-ammonia control digester) and SAO3 (high-ammonia experimental digester) traced by 16 s rRNA gene profiling using terminal restriction fragment length polymorphism (T-RFLP). Terminal restriction fragments (T-RFs) that could be affiliated to recovered genotypes are labelled by fragment size (bp) and accession number. T-RFs were grouped into stable fragments (blue), fading fragments (green), establishing fragments up to 0.62 g NH_3_/L (orange) and establishing fragments up to 0.96 g NH_3_/L (red) compared with a control digester. Fragments marked in light green were established during the time course of the control, but were still fading in the experimental digester. Peaks that emerged non-chronologically on one or two occasions are marked in black. Days of operation are plotted on the x-axis, relative peak abundance on the y-axis. 16 s rRNA gene T-RFLP data sets were collected as triplicates from each sampling point mentioned above.
10.1186/s13068-016-0454-9 Taxonomic distribution of OTUs at a threshold value of 97 %.
10.1186/s13068-016-0454-9 Abundance of methanogens, syntrophic acetate oxidisers and total bacteria in the digester samples used for the microbial analysis in the present study.
10.1186/s13068-016-0454-9 Operational data for the different digesters used for the microbial analysis.
10.1186/s13068-016-0454-9 Rarefaction analysis of the recovered 16 sRNA gene pool obtained from the experimental digester SAO1 and the control digester SAO1. Sampling points day 70 (.1), day 141 (.2), day 225 (.3), day 442 (.5), day 642 (.7).

## Abbreviations

SAOB: syntrophic acetate-oxidising bacteria
SAO: syntrophic acetate oxidation
SRA: sequence Read Archive
OTU: operational taxonomic unit
T-RFLP: terminal restriction length polymorphism
QIIME: quantitative Insights into Microbial Ecology

## References

[1] Mackie RI, Bryant MP (1981). Metabolic activity of fatty acid-oxidizing bacteria and the contribution of acetate, propionate, butyrate, and CO_2_ to methanogenesis in cattle waste at 40 and 60 °C. Appl Environ Microbiol.

[2] Mountfort DO, Asher RA (1978). Changes in proportions of acetate and carbon dioxide used as methane precursors during the anaerobic digestion of bovine waste. Appl Environ Microbiol.

[3] Zinder SH, Koch M (1984). Non-aceticlastic methanogenesis from acetate: acetate oxidation by a thermophilic syntrophic coculture. Arch Microbiol.

[4] Hattori S (2008). Syntrophic acetate-oxidizing microbes in methanogenic environments. Microbes Environ..

[5] Sprott GD, Patel GB (1986). Ammonia toxicity in pure cultures of methanogenic bacteria. Syst Appl Microbiol.

[6] Steinhaus B, Garcis ML, Shen AQ, Angenent LT (2007). A portable anaerobic microbioreactor reveals optimum growth conditions for the methanogen Methanosaeta concilii. Appl Environ Microbiol.

[7] Schnürer A, Nordberg A (2008). Ammonia, a selective agent for methane production by syntrophic acetate oxidation at mesophilic temperature. Water Sci Technol.

[8] Schnürer A, Zellner G, Svensson BH (1999). Mesophilic syntrophic acetate oxidation during methane formation in biogas reactors. FEMS Microbiol Ecol.

[9] Westerholm M, Levén L, Schnürer A (2012). Bioaugmentation of syntrophic acetate-oxidising culture in biogas reactors exposed to increasing levels of ammonia. Appl Environ Microbiol.

[10] Angenent LT, Sung S, Raskin L (2002). Methanogenic population dynamics during startup of a full-scale anaerobic sequencing batch reactor treating swine waste. Water Res.

[11] Shimada T, Morgenroth E, Tandukar M, Pavlostathis SG, Smith A, Raskin L, Kilian RE (2011). Syntrophic acetate oxidation in two-phase (acid-methane) anaerobic digesters. Water Sci Technol.

[12] Sun L, Müller B, Westerholm M, Schnürer A (2014). Syntrophic acetate oxidation in industrial CSTR biogas digesters. J Biotechnol.

[13] Fotidis IA, Karakashev D, Angelidaki I (2014). The dominant acetate degradation pathway/methanogenic composition in full-scale anaerobic digesters operating under different ammonia levels. Int J Environ Sci Technol.

[14] Ahring BK, Schmidt JE, Winther-Nielsen M, Macarion AJL, Conway de Macario E (1993). Effect of the medium composition and sludge removal on the production, composition and architecture of thermophilic (55 °C) acetate-utilizing granules from an upflow anaerobic sludge blanket reactor. Appl Environ Microbiol.

[15] Hao L, Lü F, He P, Li L, Shao L (2011). Predominant contribution of syntrophic acetate oxidation to thermophilic methane formation at high acetate concentrations. Environ Sci Technol.

[16] Karakashev D, Batstone DJ, Trably E, Angelidaki I (2006). Acetate oxidation is the dominant methanogenic pathway from acetate in the absence of *Methanosaetaceae*. Appl Environ Microbiol.

[17] Petersen SP, Ahring BK (1991). Acetate oxidation in a thermophilic anaerobic sludge-digestor: the importance of non-acetoclastic methanogenesis from acetate. FEMS Microbiol Ecol.

[18] Shigematsu T, Tang Y, Kobayashi T, Kawaguchi H, Morimura S, Kida K (2004). Effect of dilution rate on metabolic pathway shift between aceticlastic and nonacetoclastic methanogenesis in chemostat cultivation. Appl Environ Microbiol.

[19] Mayumi D, Dolfing J, Sakata S, Maeda H, Miyagawa Y, Ikarashi M, Tamaki H, Takeuchi M, Nakatsu CH, Kamagata Y (2013). Carbon dioxide concentration dictates alternative methanogenic pathways in oil reservoirs. Nat Commun.

[20] Balk M, Weijma J, Stams AJM (2002). *Thermotoga lettingae* sp. nov., a novel thermophilic, methanol-degrading bacterium isolated from a themophilic anaerobic reactor. Int J Syst Evol Microbiol.

[21] Hattori S, Kamagata Y, Hanada S, Shoun H (2000). *Thermacetogenium phaeum* gen. nov., sp. nov., a strictly anaerobic, thermophilic, syntrophic acetate-oxidizing bacterium. Int J Syst Evol Microbiol.

[22] Schnürer A, Schink B, Svensson BH (1996). *Clostridium ultunense* sp. nov., a mesophilic bacterium oxidizing acetate in syntrophic association with a hydrogenotrophic methanogenic bacterium. Int J Syst Bacteriol.

[23] Westerholm M, Roos S, Schnürer A (2010). *Syntrophaceticus schinkii* gen. nov., sp. nov., an anaerobic, syntrophic acetate-oxidizing bacterium isolated from a mesophilic anaerobic filter. FEMS Microbiol Lett.

[24] Westerholm M, Roos S, Schnürer A (2011). *Tepidanaerobacter acetatoxydans* sp. nov., an anaerobic, syntrophic acetate-oxidizing bacterium isolated from two ammonium-enriched mesophilic methanogenic processes. Syst Appl Microbiol.

[25] Drake HL, Küsel K, Matthies C (2006). Acetogenic prokaryotes.

[26] Hattori S, Galushko AS, Kamagata Y, Schink B (2005). Operation of the CO dehydrogenase/acetyl coenzyme A pathway in both acetate oxidation and acetate formation by the syntrophically acetate-oxidizing bacterium *Thermacetogenium phaeum*. J Bacteriol.

[27] Oehler D, Poehlein A, Leimbach A, Müller N, Daniel R, Gottschalk G, Schink B (2012). Genome-guided analysis of physiological and morphological traits of the fermentative acetate oxidizer *Thermacetogenium phaeum*. BMC Genomics.

[28] Schnürer A, Svensson BH, Schink B (1997). Enzyme activities in and energetics of acetate metabolism by the mesophilic syntrophically acetate-oxidizing anaerobe *Clostridium ultunense*. FEMS Microbiol Lett.

[29] Müller B, Manzoor S, Niazi A, Bongcam-Rudloff E, Schnürer A (2015). Genome-guided analysis of physiological capacities of *Tepidanaerobacter acetatoxydans* provides insights into environmental adaptations and syntrophic acetate oxidation. PLoS ONE.

[30] Nobu MK, Narihiro T, Rinke C, Kamagata Y, Tringe SG, Woyke T, Liu W (2015). Microbial dark matter ecogenomics reveals complex synergistic network in a methanogenic bioreactor. ISME J.

[31] Müller B, Sun L, Schnürer A (2012). First insights into the syntrophic acetate-oxidizing bacteria—a genetic study. MicrobiologyOpen.

[32] Leaphart AB, Lovell CR (2001). Recovery and analysis of formyltetrahydrofolate synthetase gene sequences from natural populations of acetogenic bacteria. Appl Environ Microbiol.

[33] Henderson G, Naylor GE, Leahy SC, Janssen PH (2010). Presence of novel, potentially homoacetogenic bacteria in the rumen as determined by analysis of formyltetrahydrofolate synthetase sequences from ruminants. Appl Environ Microbiol.

[34] Matsui H, Kojima N, Tajima K (2008). Diversity of the formyltetrahydrofolate synthetase gene (*fhs*), a key enzyme for reductive acetogenesis, in the bovine rumen. Biosci Biotechnol Biochem.

[35] Leaphart AB, Friez MJ, Lovell CR (2003). Formyltetrahydrofolate synthetase sequences from salt marsh plant roots reveal a diversity of acetogenic bacteria and other bacterial functional groups. Appl Environ Microbiol.

[36] Ottesen EA, Leadbetter JR (2010). Diversity of formyltetrahydrofolate synthetases in the guts of the wood-feeding cockroach *Cryptocercus punctulatus* and the omnivorous cockroach *Periplaneta americana*. Appl Environ Microbiol.

[37] Ottesen EA, Leadbetter JR (2011). Formyltetrahydrofolate synthetase gene diversity in the guts of higher termites with different diets and lifestyles. Appl Environ Microbiol.

[38] Pester M, Brune A (2006). Expression profiles of *fhs* (FTHFS) genes support the hypothesis that spirochaetes dominate reductive acetogenesis in the hindgut of lower termites. Environ Microbiol.

[39] Salmassi TM, Leadbetter JR (2003). Analysis of genes of tetrahydrofolate-dependent metabolism from cultivated spirochaetes and the gut community of the termite *Zootermopsis angusticollis*. Microbiol.

[40] Hori T, Sasaki D, Haruta S, Shigematsu T, Ueno Y, Ishii M, Igarashi Y (2011). Detection of active, potentially acetate-oxidizing syntrophs in an anaerobic digester by flux measurement and formyltetrahydrofolate synthetase (FTHFS) expression profiling. Microbiology.

[41] Ryan P, Forbes C, Colleran E (2008). Investigation of the diversity of homoacetogenic bacteria in mesophilic and thermophilic anaerobic sludges using the formyltetrahydrofolate synthetase gene. Water Sci Technol.

[42] Ryan P, Forbes C, McHugh S, O’Reilly C, Fleming GTA, Colleran E (2010). Enrichment of acetogenic bacteria in high rate anaerobic reactors under mesophilic and thermophilic conditions. Water Res.

[43] Westerholm M, Müller B, Arthurson V, Schnürer A (2011). Changes in the acetogenic population in a mesophilic anaerobic digester in response to increasing ammonia concentration. Microbes Environ.

[44] Xu K, Liu H, Du G, Chen J (2009). Real-time PCR assays targeting formyltetrahydrofolate synthetase gene to enumerate acetogens in natural and engineered environmants. Anaerobe.

[45] Gagen EJ, Denman SE, Padmanabha J, Zadbuke S, Al Jassim R, Morrision M, McSweeney CS (2010). Functional gene analysis suggests different acetogen populations in the bovine rumen and tammar wallaby forestomach. Appl Environ Microbiol.

[46] Westerholm M, Dolfing J, Sherry A, Gray ND, Head IM, Schnürer A (2011). Quantification of syntrophic acetate-oxidizing microbial communities in biogas processes. Environ Microbiol Rep.

[47] Moestedt J, Müller B, Westerholm M, Schnürer A. Ammonia threshold for inhibition of anaerobic digestion of thin stillage and the importance of organic loading rate. Microb Biotechnol. 2015. doi:10.1111/1751-7915.12330. 10.1111/1751-7915.12330 PMC4767286 26686366

[48] Westerholm M, Müller B, Isaksson S, Schnürer A (2015). Trace element and temperature effects on microbial communities and links to biogas digester performance at high ammonia levels. Biotechnol Biofuel.

[49] Snauwaert I, Stragier P, De Vuyst L, Vandammel P (2015). Comparative genomw analysis of *Pediococcus damnosus* LMG 28219, a strain well-adapted to the beer environment. BMC Genom.

[50] Hädrich A, Heuer VB, Herrmann M, Hinrichs K, Küsel K (2012). Origin and fate of acetate in an acidic fen. FEMS Microbiol Ecol.

[51] Hunger S, Schmidt O, Hilgarth M, Horn MA, Kolb S, Conrad R, Drake HL (2011). Competing formate- and carbon dioxide-utilizing prokaryotes in an anoxic methane-emitting fen soil. Appl Environ Microbiol.

[52] Lever MA, Heuer VB, Morono Y, Masui N, Schmidt F, Alperin MJ, Inagaki F, Hinrichs K, Teske A (2010). Acetogenesis in deep subseafloor sediments of the Juan de Fuca Ridge flank: a synthesis of geochemical, thermodynamic, and gene-based evidence. Geomicrobiol J.

[53] Matsui H, Yoneda S, Ban-Tokuda T, Wakita M (2011). Diversity of the formyltetrahydrofolate synthetase (FTHFS) gene in the proximal and mid ostrich colon. Curr Microbiol.

[54] Werner JJ, Garcia ML, Perkins SD, Yarasheski KE, Smith SR, Muegge B, Stadermann FJ, DeRito CM, Floss C, Madsen EL (2014). Microbial community dynamics and stability during an ammonia-induced shift to syntrophic acetate oxidation. Appl Environ Microbiol.

[55] Hahnke S, Maus I, Wibber D, Tomazetto G, Pühler A, Klocke M, Schlüter A (2015). Complete genome sequence of the novel *Porphyromonadaceae* bacterium strain ING2-E5B isolated from a mesophilic lab-scale biogas reactor. J Biotechnol.

[56] Chen S, Dong X (2005). *Proteiniphilum acetatigenes* gen. nov., sp. nov., from a UASB reactor treating brewery wastewater. Int J Syst Evol Microbiol.

[57] Schaal KP, Yassin AF, Stackebrandt E, Balows A, Truper HG, Dworkin M, Harder W, Schleifer KH (2006). The family Actinomycetaceae: the genera *Actinomyces*, *Actinobaculum*, *Arcanobacterium*, *Varibaculum*, and *Mobiluncus*. The prokaryotes: a handbook on the biology of bacteria.

[58] Bouanane-Darenfed A, Fardeau ML, Grégoire P, Joseph M, Kebbouche-Gana S, Benayad T, Hacene H, Cayol JL, Ollivier B (2011). *Caldicoprobacter algeriensis* sp. nov. a new thermophilic anaerobic, xylanolytic bacterium isolated from an Algerian hot spring. Curr Microbiol.

[59] Bouanane-Darenfed A, Ben Hania W, Hacene H, Cayol JL, Ollivier B, Fardeau ML (2013). *Caldicoprobacter guelmensis* sp. nov., a thermophilic, anaerobic, xylanolytic bacterium isolated from a hot spring. Int J Syst Evol Microbiol.

[60] Hammes WP, Hertel C (1901). Genus I. Lactobacillus Beijerink.

[61] Francisci DD, Kougias PG, Treu L, Campanaro S, Angelidaki I (2015). Microbial diversity and dynamicity of biogas reactors due to radical changes of feedstock composition. Bioresour Technol.

[62] Sträuber H, Lucas R, Kleinsteuber S (2016). Metabolic and microbial community dynamics during the anaerobic digestion of maize silage in a two-phase process. Bioenerg Biofuel.

[63] Park D, Lee WJ, Jang I, Lee W (2015). Got Lactobacillus? Commensals power growth. Cell Host Microbe.

[64] Bassani I, Kougias PG, Treu L, Angelidaki I (2015). Biogas upgrading via hydrogenotrophic methanogenesis in two-stage continuous stirred tank reactors at mesophilic and thermophilic conditions. Environ Sci Technol.

[65] Lee S, Park J, Kim SH, Yu BJ, Yoon J, Park H (2015). Evidence off syntrophic acetate oxidation by *Spirochaetes* during anaerobic methane production. Bioresour Technol.

[66] Levén L, Eriksson A, Schnürer A (2007). Effect of process temperature on bacterial and archaeal communities in two methanogenic bioreactors treating organic household waste. FEMS Microbiol Ecol.

[67] Rozen S, Skaletsky H (2000). Primer3 on the WWW for general users and for biologist programmers. Methods Mol Biol.

[68] Katoh K, Kuma K, Toh H, Miyata T (2005). MAFFT version 5: improvement in accuracy of multiple sequence alignment. Nucliec Acids Res.

[69] Guindon S, Gascuel O (2003). A simple, fast and accurate algorithm to estimate large phylogenies by maximum Likelihood. Syst Biol.

[70] Hugerth LW, Wefer HA, Lundin S, Jakobsson HE, Lindberg M, Rodin S, Engstrand L, Andersson AF (2014). DegePrime, a program for degenerate primer design for broad-taxonomic range PCR in microbial ecology studies. Appl Environ Microbiol.

[71] Martin M. Cutadapt removes adapter sequences from high-throughput sequencing reads. EMBnet.journal. 2011;17:10–2.

[72] Caporaso JG, Kuczynski J, Stombaugh J, Bittinger K, Bushman FD, Costello EK, Fierer N, Pena AG, Goodrich JK, Gordon JI (2010). QIIME allows analysis of high-throughput community sequencing data. Nat Methods.

[73] Rideout JR, He Y, Navas-Molina JA, Walters WA, Ursell LK, Gibbons SM, Chase J, McDonald D, Gonzalez A, Robbins-Pianka A (2014). Subsampled open-reference clustering creates consistent, comprehensive OTU definitions and scales to billions of sequences. PeerJ.

[74] Edgar RC (2010). Search and clustering orders of magnitude faster than BLAST. Bioinformatics.

[75] Caporaso JG, Bittinger K, Bushman FD, DeSantis TZ, Andersen GL, Knight R. PyNAST: a flexible tool for aligning sequences to a template alignment. Bioinformatics. 2010;26:266–7. 10.1093/bioinformatics/btp636 PMC2804299 19914921

[76] Haas BJ, Gevers D, Earl AM, Feldgarden M, Ward DV, Giannoukos G, Ciulla D, Tabbaa D, Highlander SK, Sodergren E. Chimeric 16S rRNA sequence formation and detection in Sanger and 454-pyrosequenced PCR amplicons. Genome Res. 2011;21:494–504. 10.1101/gr.112730.110 PMC3044863 21212162

[77] Wang Q, Garrity GM, Tiedje JM, Cole JR. Naïve Bayesian classifier for rapid assignment of rRNA sequences into the new bacterial taxonomy. Appl Environ Microbiol. 2007;73:5261–7. 10.1128/AEM.00062-07 PMC1950982 17586664

[78] Price MN, Dehal PS, Arkin AP. FastTree 2—approximately maximum-likelihood trees for large alignments. PLoS One. 2010;5:e9490. 10.1371/journal.pone.0009490 PMC2835736 20224823

