# Supplementary material for: Bacterial community composition and fhs profiles of low- and high-ammonia biogas digesters reveal novel syntrophic acetate-oxidising bacteria
Source: Biotechnol Biofuels. 2016 Feb 27;9:48. doi: 10.1186/s13068-016-0454-9 (PMC4769498; doi:10.1186/s13068-016-0454-9)
Supplement: Supplementary file 2 — 10.1186/s13068-016-0454-9 Summary of qPCR amplification efficiency and primer specificity. OTU primer specificity was confirmed by standard PCR using selected industrial DNA samples as listed in the table. [file 13068_2016_454_MOESM2_ESM.docx]

Table S1: Summary of qPCR amplification efficiency and primer specificity. OTU primer specificity was confirmed by standard PCR using selected industrial DNA samples as listed in the table.

| OTU | Efficiency E [%] | Linear regression R”2 | Slope | Primer specificity confirmed in sample: |
| --- | --- | --- | --- | --- |
| OTU3 | 103.9 | 0.972 | -3.233 | D/E/G/H/J |
| OTU4 | 76.0 | 0.994 | -4.072 | F/G/H |
| OTU5 | 120.1 | 0.965 | -2.918 | D/E/G |
| OTU6 | 85.8 | 0.997 | -3.718 | M/L/G |
| OTU7 | 104.1 | 0.995 | -3.228 | G/J |
| OTU8 | 117.0 | 0.938 | -2.973 | D/E/F/G/H/J |
| OTU9 | 88.3 | 0.997 | -3.637 | D/E/G/J |
| OTU10 | 95.8 | 0.979 | -3.428 | D/F/H/G |
