# Supplementary material for: Bacterial community composition and fhs profiles of low- and high-ammonia biogas digesters reveal novel syntrophic acetate-oxidising bacteria
Source: Biotechnol Biofuels. 2016 Feb 27;9:48. doi: 10.1186/s13068-016-0454-9 (PMC4769498; doi:10.1186/s13068-016-0454-9)
Supplement: Supplementary file 3 — 10.1186/s13068-016-0454-9 Summary of OTU gene copy numbers in log scale. [file 13068_2016_454_MOESM3_ESM.docx]

Table S2. Summary of OTU gene copy numbers in log scale

| Digesters | OTU4 | OTU3 | OTU9 | OTU10 | OTU8 | OTU5 | OTU6 | OTU7 |
| --- | --- | --- | --- | --- | --- | --- | --- | --- |
| B^1^ | 0 | 0 | 0 | 3.98 +/- 0.21 | 0 | 0 | 0 | 2.29 +/- 1.98 |
| C^1^ | 0 | 0 | 0 | 0 | 0 | 0 | 0 | 0 |
| D^1^ | 0 | 5.55 +/- 0.10 | 4.66 +/-0.07 | 4.13 +/- 0.10 | 6.17 +/- 0.20 | 6.80 +/- 0.15 | 0 | 4.59 +/-0.4 |
| E^1^ | 0 | 5.91 +/- 0.29 | 4.54 +/-0.19 | 2.61 +/- 2.27 | 6.56 +/- 0.27 | 6.84 +/- 0.14 | 0 | 0 |
| H^1^ | 5.94 +/- 0.11 | 5.92 +/- 0.02 | 4.47 +/-0.70 | 4.64 +/- 0.12 | 6.15 +/-0.09 | 7.79 +/- 0.05 | 0 | 4.08 +/-0.32 |
| F^1^ | 7.10 +/- 0.13 | 0 | 0 | 2.27 +/- 2.35 | 5.97 +/- 0.16 | 0 | 0 | 1.7 +/-2.94 |
| G^1^ | 8.37 +/- 0.568 | 7.68 +/- 0.46 | 6.62 +/-0.47 | 1.38 +/- 2.4 | 6.33 +/- 0.57 | 9.88 +/- 1.00 | 5.15 +/- 0.52 | 7.5 +/- 0.08 |
| J^1^ | 7.35 +/- 0.23 | 7.06 +/- 0.11 | 5.64 +/-0.04 | 4.09 +/-0.10 | 5.79 +/- 0.09 | 7.22 +/- 0.08 | 0 | 5.51 +/-0.69 |
| L^1^ | 0 | 0 | 0 | 2.51 +/- 1.77 | 0 | 0 | 6.00 +/- 0.03 | 3.01 +/-2.12 |
| M^1^ | 0 | 3.53 +/-0.03 | 0 | 2.68 +/- 2.33 | 0 | 0 | 5.18 +/- 0.21 | 0 |
| SAO3^2^ Day 442 | 8.0 +/- 0.13 | 6.97 +/-0.24 | 7.45 +/-0.1 | 8.24 +/-0.9 | 6.66 +/- 0.17 | 8.44 +/- 0.14 | 4.95 +/- 0.07 | 7.76 +/- 0.13 |
| R1^3^ Day390 | 0 | 0 | 0 | 6.97 | 5.84 | 5.68 | 0 | 6.33 |
| R2^3^  Day390 | 0 | 0 | 0 | 6.93 | 6.5 | 6.8 | 0 | 7.15 |

Data from ^1^ Commercial scale, Sun et al. 2014; ^2^ Lab-scale, Schnürer and Nordberg, 2008; ^3^ Lab-scale^,^ Westerholm et al. 2012; ^3^ DNA samples were not analyzed in replicates due to the experimental duplicate ( parallel lab-scale digesters operated with same conditions)
