# Supplementary material for: Bacterial community composition and fhs profiles of low- and high-ammonia biogas digesters reveal novel syntrophic acetate-oxidising bacteria
Source: Biotechnol Biofuels. 2016 Feb 27;9:48. doi: 10.1186/s13068-016-0454-9 (PMC4769498; doi:10.1186/s13068-016-0454-9)
Supplement: Supplementary file 5 — 10.1186/s13068-016-0454-9 Summary of the partial fhs sequences and OTUs obtained, the in silico T-RFLP fragment and the closest relative. [file 13068_2016_454_MOESM5_ESM.docx]

Table S3: Summary of the partial *fhs* sequences and OTUs obtained, the in-silico T-RFLP fragment, and the closest relative identified.

| **Accession number or OTU** | **T-RFLP fragment**  **(*Hpy*188III)** | **Digester** | **Suggested**  **cluster** | **Highest amino acid sequence identity to** |
| --- | --- | --- | --- | --- |
|  |  |  |  |  |
|  |  |  |  |  |
| OTU1 | 635bp | SAO3 |  | 81% *Caloranaerobacter azorensis (Clostridiaceae)* |
| OTU2 | 635bp | SAO3 |  | 98% *Tepidanaerobacter acetatoxydans (Thermoanaerobacteraceae)* |
| OTU3 | 635bp | SAO3 | pSAOB cluster II | 78% *Moorella thermoacetica (Thermoanaerobacteraceae)* |
| OTU4 | 298bp | SAO3 |  | 84% *Enterococcus pallens (Enterococcaceae)* |
| OTU5 | 635bp | SAO3 | pSAOB cluster I | 79% *Mahella australiensis (Thermoanaerobacterales Family IV)* |
| OTU6 | 635bp | SAO3 |  | 82 % *Pseudoflavonifractor capillosus (Clostridiales)* |
| OTU7 | 635bp | SAO3 | pSAOB cluster III | 90% *Clostridium ultunense (Clostridiaceae)* |
| OTU8 | 283bp | SAO3 |  | 83% *Pseudobacteroides cellulosolvens (Ruminococcaceae)* |
| OTU9 | 378bp | SAO3 | pSAOB cluster I | 87% *Peptococcaceae bacterium* 1109 *(Peptococcaceae)* |
| OTU10 | 378bp | SAO3 |  | *Pediococcus damnosus (Lactobacillaceae)* |
| JQ082256 | 635bp | SAO3 | pSAOB cluster II | 76% *Ruminococcus flavefaciens (Ruminococcaceae)* |
| JQ082259 | 91bp | SAO3 |  | 100% *Aminobacterium colombiense (Synergistaceae)* |
| JQ082260 | 470bp | SAO3 |  | 85% *Thermanaerobacter kivui (Thermoanaerobacteraceae)* |
| JQ082261 | 91bp | SAO3 |  | 85% *Thermanaerobacter kivui (Thermoanaerobacteraceae)* |
| JQ082266 | 635bp | SAO3 |  | 88% *Eubacterium infirmum (Clostridiales)* |
| JQ082273 | 493bp | SAO3 |  | 99% *Tepidanaerobacter acetatoxydans (Thermoanaerobacteraceae)* |
| JQ082275 | 589bp | SAO3 | pSAOB cluster I | 74% *Mahella australiensis (Thermoanaerobacterales* Family IV*)* |
| JQ082276 | 91bp | SAO3 | pSAOB cluster I | 82% *Mahella australiensis (Thermoanaerobacterales* Family IV*)* |
| JQ082278 | 582bp | SAO3 |  | 82% *Mahella australiensis (Thermoanaerobacterales* Family IV*)* |
| JQ082283 | 91bp | SAO3 | pSAOB cluster I | 85% *Peptococcaceae bacterium* 1109 |
| JQ082286 | 310bp | SAO3 | pSAOB cluster III | 91% *Clostridium ultunense (Clostridiaceae)* |
| JQ082295 | 382bp | SAO3 | pSAOB cluster II | 78% *Moorella glycerini (Thermoanaerobacteraceae)* |
| JQ240268 | 283bp | SAO1 |  | 69% *Clostridium sp.* CAG:288 *(Clostridiaceae)* |
| JQ240269 | 270bp | SAO1 |  | 92% *Phascolarctobacterium succinatutens (Selenomonadales;*  *Acidaminococcaceae)* |
| JQ240270 | 302bp | SAO1 |  | 76% *Ruminococcus bromii, (Ruminococcaceae)* |
| JQ240271 | 635bp | SAO1 |  | 88% *Syntrophomonas zehnderi (Syntrophomonadaceae;*  *Syntrophomonas)* |
| JQ24072 | 635bp | SAO1 | AD cluster I | 69% *Thermanaerobacter kivui (Thermoanaerobacteraceae)* |
| OTU11 | 86bp | SAO1 | AD cluster II | 93% *Sedimentibacter spec.* B4 *(Clostridiales, Sediminibacter)* |
| OTU12 | 635bp | SAO1 | AD cluster I | 71% *Thermanaerobacter kivui (Thermoanaerobacteraceae)* |
| OTU13 | 86bp | SAO1 |  | 80% *Moorella thermoacetica (Thermoanaerobacteraceae)* |
| OTU14 | 86bp | SAO1 | AD cluster II | 91% *Sedimentibacter spec.* B4 *(Clostridiales, Sediminibacter)* |
| OTU15 | 635bp | SAO1 |  | 88% *Eubacterium infirmum (Clostridiales)* |
| OTU16 | 159bp | SAO1 | AD cluster I | 71% *Pyramidobacter piscolens (Synergistaceae)* |
| OTU17 | 635bp | SAO1 | AD cluster I | 70% *Thermanaerobacter kivui (Thermoanaerobacteraceae)* |
| OTU18 | 50bp | SAO1 |  | 80% *Pseudobacteroides cellulosolvens (Ruminococcaceae)* |
| OTU19 | 477bp | SAO1 | AD cluster III | 80% *Odoribacter laneus (Porhyromonadaceae)* |
| OTU20 | 593bp | SAO1 | AD cluster III | 79% *Odoribacter laneus (Porhyromonadaceae)* |
| JQ082214 | 297bp | SAO1 |  | 79% *Thermanaerobacter kivui (Thermoanaerobacteraceae)* |
| JQ082217 | 226 bp | SAO1 |  | 87% *Holdemanella biformes (Erysipelotrichaceae)* |
| JQ082219 | 84bp | SAO1 | AD cluster III | 79% *Cecembia lonarensis (Cyclobactericaea)* |
| JQ082223 | 635bp | SAO1 |  | 85% *Syntrophomonas zehnderi (Syntrophomonadaceae;*  *Syntrophomonas)* |
| JQ082227 | 297bp | SAO1 |  | 75% *Anaeromusa acidaminophila (Selenomonadales;Veillonellaceae)* |
| JQ082233 | 342bp | SAO1 |  | 89% *Synergistis jonesii (Synergistaceae)* |
| JQ082237 | 635bp | SAO1 |  | 73% *Tepidanaerobacter acetatoxydans (Thermoanaerobacteraceae)* |
| JQ082238 | 310bp | SAO1 |  | 74% *Thermanaerobacter kivui (Thermoanaerobacteraceae)* |
| JQ082239 | 91bp | SAO1 | AD cluster I | 74% *Subdoligranulum variabile (Ruminococcaceae)* |
| JQ082241 | 635bp | SAO1 | AD cluster I | 72% *Faecalibacterium prausnitzii (Ruminococcaceae)* |
| JQ082242 | 91bp | SAO1 |  | 84% *Enorma massiliensis (Coriobacteriaceae)* |
| JQ082243 | 379bp | SAO1 | AD cluster I | 71% *Ruminococcus champanellensis (Ruminococcaceae)* |
| JQ082253 | 463bp | SAO1 |  | 78% *Odoribacter laneus (Porhyromonadaceae)* |
