# Supplementary material for: Bacterial community composition and fhs profiles of low- and high-ammonia biogas digesters reveal novel syntrophic acetate-oxidising bacteria
Source: Biotechnol Biofuels. 2016 Feb 27;9:48. doi: 10.1186/s13068-016-0454-9 (PMC4769498; doi:10.1186/s13068-016-0454-9)
Supplement: Supplementary file 6 — 10.1186/s13068-016-0454-9 Distance-matrix of the deduced amino acid sequence of recovered partial fhs sequences and known SAOB constructed by MAFFT v7.017. [file 13068_2016_454_MOESM6_ESM.docx]

Table S4: Distance-matrix of the deduced amino acid sequence of recovered partial *fhs* sequences and known SAOB constructed by MAFFT v7.017 [1]


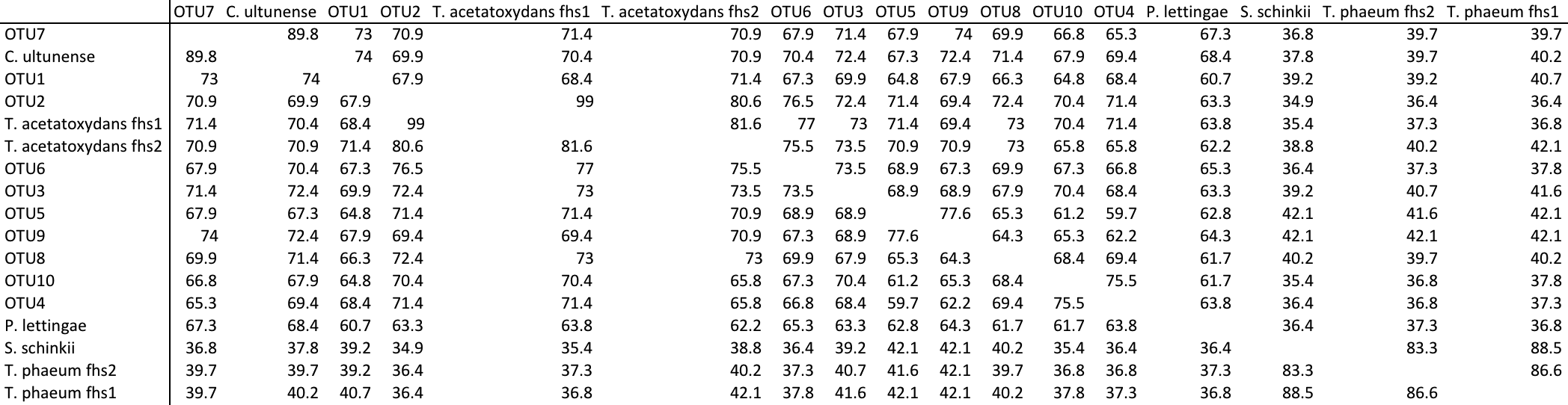


[1] Katoh K, Misawa K, Kuma K, Miyata T: **MAFFT: a novel method for rapid multiple sequence alignment based on fast Fourier transform.** *Nucleic Acids Res*. 2002, **30:**3059-3066.
