# Supplementary figures and images for: Bacterial community composition and fhs profiles of low- and high-ammonia biogas digesters reveal novel syntrophic acetate-oxidising bacteria
Source: Biotechnol Biofuels. 2016 Feb 27;9:48. doi: 10.1186/s13068-016-0454-9 (PMC4769498; doi:10.1186/s13068-016-0454-9)

**Control digester SAO1**

**Experimental digester SAO3**

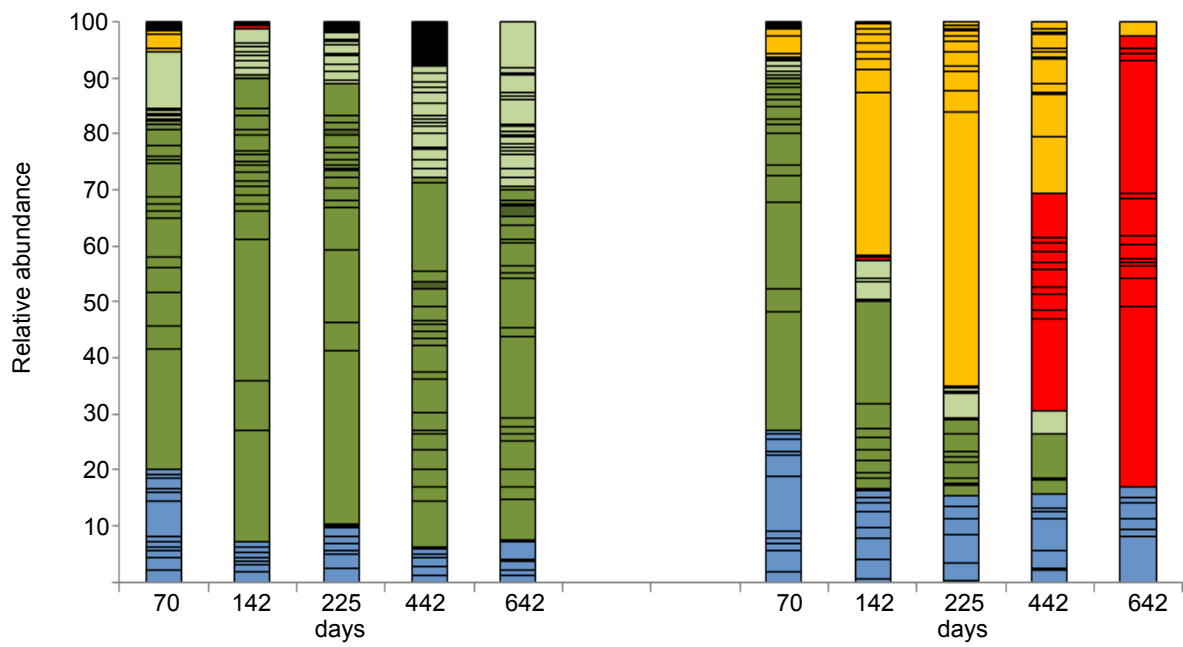

## control

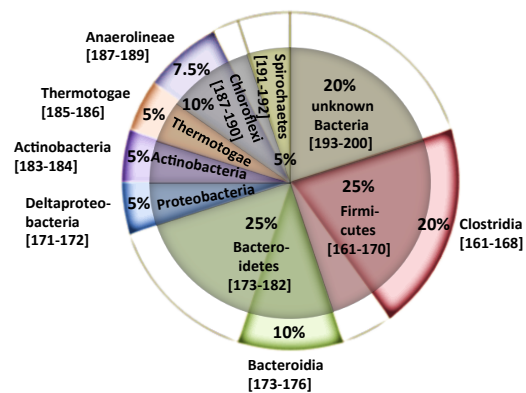

## NH<sub>4</sub><sup>+</sup>-N stressed

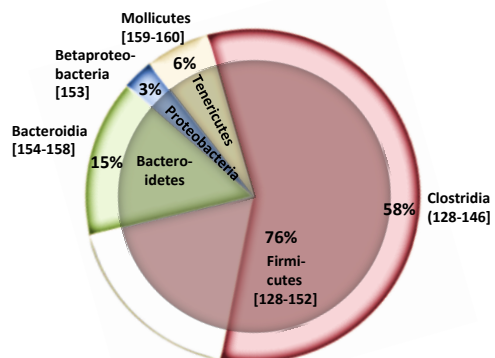

Supplement: Supplementary file 7 — 10.1186/s13068-016-0454-9 Dynamics of the bacterial community in SAO1 (low-ammonia control digester) and SAO3 (high-ammonia experimental digester) traced by 16 s rRNA gene profiling using terminal restriction fragment length polymorphism (T-RFLP). Terminal restriction fragments (T-RFs) that could be affiliated to recovered genotypes are labelled by fragment size (bp) and accession number. T-RFs were grouped into stable fragments (blue), fading fragments (green), establishing fragments up to 0.62 g NH3/L (orange) and establishing fragments up to 0.96 g NH3/L (red) compared with a control digester. Fragments marked in light green were established during the time course of the control, but were still fading in the experimental digester. Peaks that emerged non-chronologically on one or two occasions are marked in black. Days of operation are plotted on the x-axis, relative peak abundance on the y-axis. 16 s rRNA gene T-RFLP data sets were collected as triplicates from each sampling point mentioned above. [file 13068_2016_454_MOESM7_ESM.pdf]

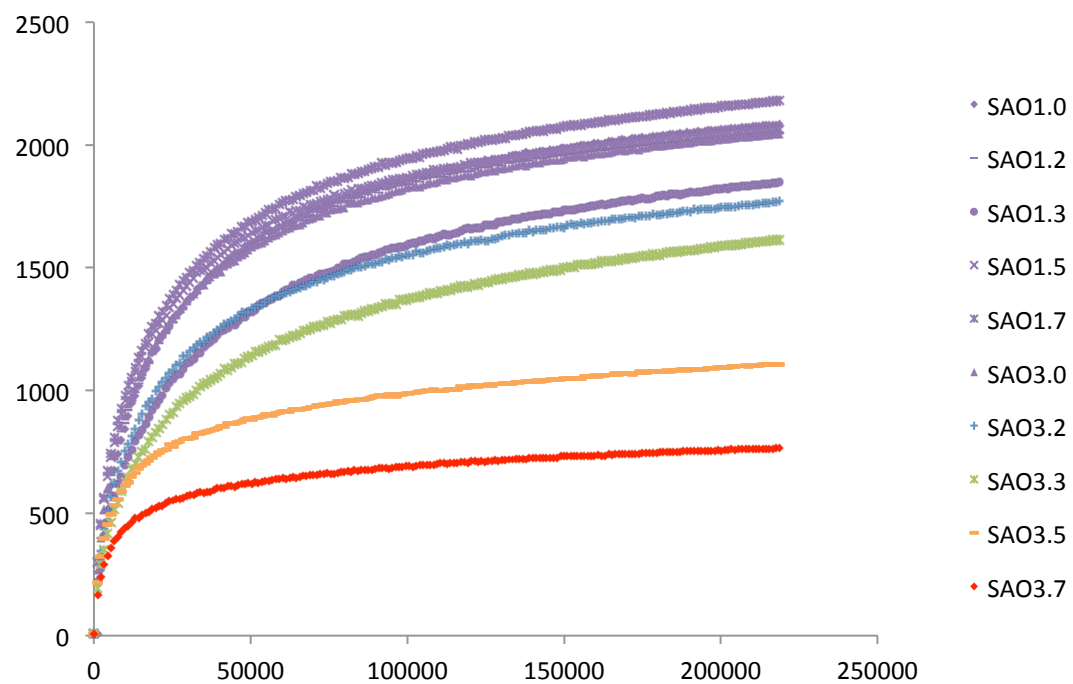

Supplement: Supplementary file 11 — 10.1186/s13068-016-0454-9 Rarefaction analysis of the recovered 16 sRNA gene pool obtained from the experimental digester SAO1 and the control digester SAO1. Sampling points day 70 (.1), day 141 (.2), day 225 (.3), day 442 (.5), day 642 (.7). [file 13068_2016_454_MOESM11_ESM.pdf]
