# Supplementary material for: Bacterial community composition and fhs profiles of low- and high-ammonia biogas digesters reveal novel syntrophic acetate-oxidising bacteria
Source: Biotechnol Biofuels. 2016 Feb 27;9:48. doi: 10.1186/s13068-016-0454-9 (PMC4769498; doi:10.1186/s13068-016-0454-9)
Supplement: Supplementary file 9 — 10.1186/s13068-016-0454-9 Abundance of methanogens, syntrophic acetate oxidisers and total bacteria in the digester samples used for the microbial analysis in the present study. [file 13068_2016_454_MOESM9_ESM.docx]

Table S6: Abundance of methanogens, syntophic acetate oxidisers and total bacteria in the digester samples used for the microbial analysis in the present study. Logarithmic gene copies/mL of *Methanomicrobiales (MMB)*, *Methanobacteriales (MBT)*, *Methanosarcinaceae (MSc), Methanosaeta (Mst), Clostridium ultunese (Cult), Tepidanaerobacter acetatoxydans (Tp), Syntrophaceticus schinkii (THAC) and Methanoculleus bourgensis (MAB).*

| Digesters | MMB | MBT | Mst | Msc | MAB | Cult | Tp | THAC | Total  bacteria |
| --- | --- | --- | --- | --- | --- | --- | --- | --- | --- |
| B^1^ | 8.2 | 7.6 | 8.4 | 6.7 | ND | 0^a^ | 0^a^ | 7.1 | 12 |
| C^1^ | 7.8 | 8.0 | 6.2 | 9.0 | ND | 0^a^ | 0^a^ | 6.2 | 13 |
| D^1^ | 7.6 | 7.6 | 6.5 | 4.2 | ND | 0^a^ | 0^a^ | 11 | 12 |
| E^1^ | 7.8 | 8.0 | 5.8 | 4.6 | ND | 0^a^ | 5.3 | 11 | 12 |
| H^1^ | 9.2 | 7.2 | 5.6 | 5.7 | ND | 4.8 | 6.1 | 10 | 12 |
| F^1^ | 6.3 | 8.7 | 6.4 | 7.3 | ND | 0^a^ | 4.5 | 7.3 | 12 |
| G^1^ | 7.1 | 6.5 | 6.6 | 5.6 | ND | 7.2 | 7.2 | 11 | 13 |
| J^1^ | 7.0 | 4.1 | 5.3 | 4.4 | ND | 6.9 | 6.5 | 10 | 13 |
| L^1^ | 6.7 | 10 | 0^a^ | 4.7 | ND | 0^a^ | 5.3 | 6.8 | 13 |
| M^1^ | 8.8 | 9.1 | 0^a^ | 6.4 | ND | 0^a^ | 4.0 | 7.1 | 13 |
|  |  |  |  |  |  |  |  |  |  |
| SAO1^2^ Day 70-642 | 7.8-8.5 | ND | 7.9-9.3 | 7.0-7.4 | ND | 5.5-5.7 | 3.1-4.4 | 5.4-6.7 | 10.8-11.3 |
| SAO3^2^ Day 70 | 8.3 | ND | 6.7 | 7.5 | ND | 5.7 | 3.1 | 6.0 | 10.9 |
| SAO3^2^ Day 141 | 6.2 | ND | 5.7 | 8.9 | ND | 5.8 | 3.8 | 6.0 | 10.9 |
| SAO3^2^ Day 225 | 6.2 | ND | 4.7 | 9.1 | ND | 5.4 | 3.9 | 6.1 | 10.6 |
| SAO3^2^ Day 442 | 6.9 | ND | 5.4 | 7.9 | ND | 7.4 | 9.7 | 9.3 | 11.3 |
| SAO3^2^ Day 642 | 7.9 | ND | 2.3 | 6.1 | ND | 7.1 | 8.1 | 8.8 | 11.1 |
|  |  |  |  |  |  |  |  |  |  |
| R1^3^ Day 63-184 | 8.7-11 | 7.8-8.2 | 9.8-10 | 4.6-5.7 | 7.4-8.7 | 0^a^-3.8 | 0^a^ | 7.5-8.0 | ND |
| R1^3^ Day 212 | 9.2 | 7.6 | 10 | 5.2 | 7.4 | 0^a^ | 0^a^ | 7.1 | ND |
| R1^3^ Day 253 | 11 | 8.4 | 10 | 5.3 | 8.9 | 0^a^ | 0^a^ | 7.0 | ND |
| R1^3^ Day 266 | 10 | ND | 9.9 | 4.8 | 8.6 | 0^a^ | 0^a^ | 7.1 | ND |
| R1^3^ Day 301 | 9.9 | 8.0 | 8.4 | 4.5 | 8.3 | 2.7 | 0^a^ | 8.8 | ND |
| R1^3^ Day 345 | 9,7 | 7.9 | 7.9 | 3.9 | 8.2 | 4.2 | 4.4 | 9.4 | ND |
| R1^1^day 392 | 9.7 | 8.0 | 8.0 | 4.4 | 8.3 | 6.1 | 4.6 | 9.8 | ND |
| R1^1^day 442 | 8.0 | 7.0 | 8.0 | 5.0 | 6 | 7 | 6.5 | 9.6 | ND |
| R2^3^ Day 63-184 | 8.7-11 | 7.7-8.3 | 9.8-11 | 4.7-5.7 | 6.5-7.4 | 0^a^ | 0^a^ | 7.1-7-8 | ND |
| R2^3^ Day 212 | 8.9 | 7.7 | 11 | 5.3 | 6.7 | 0^a^ | 0^a^ | 7.0 | ND |
| R2^3^ Day 253 | 10 | 7.8 | 9.6 | 5.2 | 8.3 | 0^a^ | 0^a^ | 7.1 | ND |
| R2^3^ Day 266 | 10 | ND | 9.9 | 4.6 | 8.4 | 0^a^ | 0^a^ | 7.8 | ND |
| R2^3^ Day 301 | 9.3 | 7.9 | 8.4 | 4.4 | 7.4 | 0^a^ | 0^a^ | 9.5 | ND |
| R2^3^ Day 345 | 9.5 | 7.9 | 8.4 | 4.2 | 8.0 | 0^a^ | 4.7 | 9.9 | ND |
| R2^1^day 392 | 9.9 | 7.9 | 7.8 | 4.0 | 8.3 | 6.1 | 5.1 | 9.9 | ND |
| R2^1^day 442 | 9.3 | 7.7 | 8.0 | 5.3 | 7.4 | 7.6 | 6.5 | 10 | ND |

Data from ^1^ Commercial scale, Sun et al. 2014; ^2^ Lab-scale, Schnürer and Nordberg, 2008; ^3^Lab-scale^,^ Westerholm et al. 2012; ^a^ Below detection limit (<4), Nd, not determined. Data given represents avarage values calculated from triplicate analysis (standard deviation given in original publications.)
