# Supplementary material for: Bacterial community composition and fhs profiles of low- and high-ammonia biogas digesters reveal novel syntrophic acetate-oxidising bacteria
Source: Biotechnol Biofuels. 2016 Feb 27;9:48. doi: 10.1186/s13068-016-0454-9 (PMC4769498; doi:10.1186/s13068-016-0454-9)
Supplement: Supplementary file 10 — 10.1186/s13068-016-0454-9 Operational data for the different digesters used for the microbial analysis. [file 13068_2016_454_MOESM10_ESM.docx]

Table S7. Operational data for the different digesters used for the microbial analysis

| Digesters | Main Substrate | Temp (^o^C) | TAN^a^ (g/L) | NH_3_^b^ (g/L) | pH | HRT^c^ (days) | VFA^d^ (g/L) | OLR^f^ (gVS/L/day) | ^14^CO_2_/^14^CH_4_^g^ |
| --- | --- | --- | --- | --- | --- | --- | --- | --- | --- |
| B^1^ | Sewage sludge | 38 | 2.6 | 0.09 | 7.4 | 20 | 0.12 | 2.2 | 0.11 |
| C^1^ | Cow manure | 38 | 0.9 | 0.07 | 7.4 | 130 | 0.05 | 0.1 | 0.27 |
| D^1^ | SSMSW, food industrial waste | 36 | 3.9 | 0.18 | 7.6 | 25 | 4.0 | 3.0 | 1.2 |
| E^1^ | SSMSW, food industrial waste | 37 | 3.3 | 0.16 | 7.6 | 37 | 6.0 | 2.5 | 1.6 |
| H^1^ | SSMSW, grass silage | 38 | 2.7 | 0.17 | 7.7 | 20 | 4.0 | 3.5 | 11 |
| F^1^ | Food industrial waste, grass silage | 40 | 3.5 | 0.31 | 7.8 | 101 | 3.1 | 3.0 | 3.8 |
| G^1^ | Chicken manure, food industrial waste | 37 | 5.2 | 0.25 | 7.6 | 64 | 4.7 | 3.0 | 6.2 |
| J^1^ | Sillage and wheat boss | 38 | 4.9 | 0.31 | 7.7 | 45 | 13 | 3.2 | 34 |
| L^1^ | SSMSW, slaughterhouse waste | 55 | 2.5 | 0.82 | 8.1 | 60 | 2.4 | 1.0 | 6.6 |
| M^1^ | Industrial food waste, manure | 53 | 3.2 | 0.57 | 7.8 | 24 | 3.8 | 4.0 | 6.8 |
|  |  |  |  |  |  |  |  |  |  |
| SAO1^2^ Day 70-642 | Household waste, egg albumin | 37 | 0.65-0.90 | 0.02 | 7.2-7.4 | 30 | <0.1 | 3.0 | 0.5 |
| SAO3^2^ Day 70 | Household waste, egg albumin | 37 | 0.82 | 0.02 | 7.2 | 30 | <0.1 | 3.0 | 0.5 |
| SAO3^2^ Day 141 | Household waste, egg albumin | 37 | 1.9 | 0.09 | 7.6 | 30 | 4.0 | 3.0 | 0.5 |
| SAO3^2^ Day 225 | Household waste, egg albumin | 37 | 3.3 | 0.30 | 7.9 | 30 | 18 | 3.0 | 0.5 |
| SAO3^2^ Day 442 | Household waste, egg albumin | 37 | 5.5 | 0.62 | 8.0 | 30 | ND | 3.0 | 2.1 |
| SAO3^2^ Day 642 | Household waste, egg albumin | 37 | 6.9 | 0.96 | 7.9 | 30 | 30 | 3.0 | 2.5 |
|  |  |  |  |  |  |  |  |  |  |
| R1^3^ Day 0-183 | Stillage and cow manure | 37 | 1.1-1.5 | 0.05-0.06 | 7.5-7.8 | 57 | <0.1 | 0.8 | 1.2 |
| R1^3^ Day 184-305 | Stillage and cow manure | 37 | 2.4-3.2 | 0.10-0.21 | 7.6-7.8 | 26-50 | Acetate: <0.1 – 4.5  Propionate: <0.1 – 1.9 | 1.7-3.6 | 0.3 |
| R1^3^ Day 306-458 | Stillage and cow manure | 37 | 3.8-12.0 | 0.23-0.47 | 7.5-8.0 | 43-45 | Acetate: 1.4 – 16.0  Propionate: 2.7 – 10.0 | 2.4-2.5 | 1.8 |
| R2^3^ Day 0-183 | Stillage and cow manure | 37 | 1.1-1.5 | 0.04-0.07 | 7.5-7.8 | 57 | <0.1 | 0.8 | 1.2 |
| R2^3^ Day 184-305 | Stillage and cow manure | 37 | 2.4-3.3 | 0.14-0.23 | 6.5-7.0 | 26-50 | Acetate: <0.1-2.0  Propionate: <0.1-0.3 | 1.7-3.6 | 0.3 |
| R2^3^  Day 306-458 | Stillage and cow manure | 37 | 3.8-10.9 | 0.23-0.81 | 7.4-8.1 | 43-47 | Acetate: 1.3-15.0  Propionate: 0.3-9.5 | 2.4-2.5 | 1.8 |

Data from ^1^ Commercial scale, Sun et al. 2014; ^2^ Lab-scale, Schnürer and Nordberg, 2008; ^3^ Lab-scale^,^ Westerholm et al. 2012; ^a^ Total ammonium nitrogen; ^b^ Free ammonia; ^c^ Hydraulic retention time; ^d^ Volatile fatty acids; ^e^ Organic loading rate; ^g^ ^14^C-labelling analysis. Value > 1 indicates syntrophic acetate oxidation as the dominant methane production pathway; ND, not determined. Data given represents average values from multiple sampling occasions (information about number of samples and standard deviation given in original publications.)
